# Controls on the evolution of Ediacaran metazoan ecosystems: A redox perspective

**DOI:** 10.1111/gbi.12232

**Published:** 2017-04-07

**Authors:** F. Bowyer, R. A. Wood, S. W. Poulton

**Affiliations:** ^1^ School of GeosciencesUniversity of EdinburghEdinburghUK; ^2^ School of Earth and EnvironmentUniversity of LeedsLeedsUK

## Abstract

A growing number of detailed geochemical studies of Ediacaran (635–541 Ma) marine successions have provided snapshots into the redox environments that played host to the earliest known metazoans. Whilst previous compilations have focused on the global evolution of Ediacaran water column redox chemistry, the inherent heterogeneity evident in palaeogeographically distinct environments demands a more dissected approach to better understand the nature, interactions and evolution of extrinsic controls on the development of early macrobenthic ecosystems. Here, we review available data of local‐scale redox conditions within a palaeogeographic and sequence stratigraphic framework, to explore the mechanisms controlling water column redox conditions and their potential impact on the record of metazoans. The openly connected Laurentian margin, North America (632–540 Ma) and Nama basin, Namibia (550–538 Ma), and the variably restricted Yangtze Block, South China (635–520 Ma), show continued redox instability after the first fossil evidence for metazoans. This may support opportunistic benthic colonisation during periods of transient oxygenation amidst episodic upwelling of anoxic waters beneath a very shallow, fluctuating chemocline. The first skeletal metazoans appeared under conditions of continued redox stratification, such as those which characterise the Dengying Formation of the Yangtze Block and the Kuibis Subgroup of the Nama basin. Current data, however, suggests that successful metazoan reef‐building demanded more persistent oxia. We propose that cratonic positioning and migration throughout the Ediacaran Period, in combination with gradually increasing dissolved oxygen loading, may have provided a first‐order control on redox evolution through regulating circulation mechanisms in the Mirovian Ocean. Some unrestricted lower slope environments from mid‐high latitudes benefited from sustained oxygenation via downwelling, whilst transit of isolated cratons towards more equatorial positions stifled pervasive ventilation either through ineffective surface ocean mixing, Ekman‐induced upwelling, elevated surface ocean productivity or a combination of these processes.

## 1 Introduction

**Figure 1:**
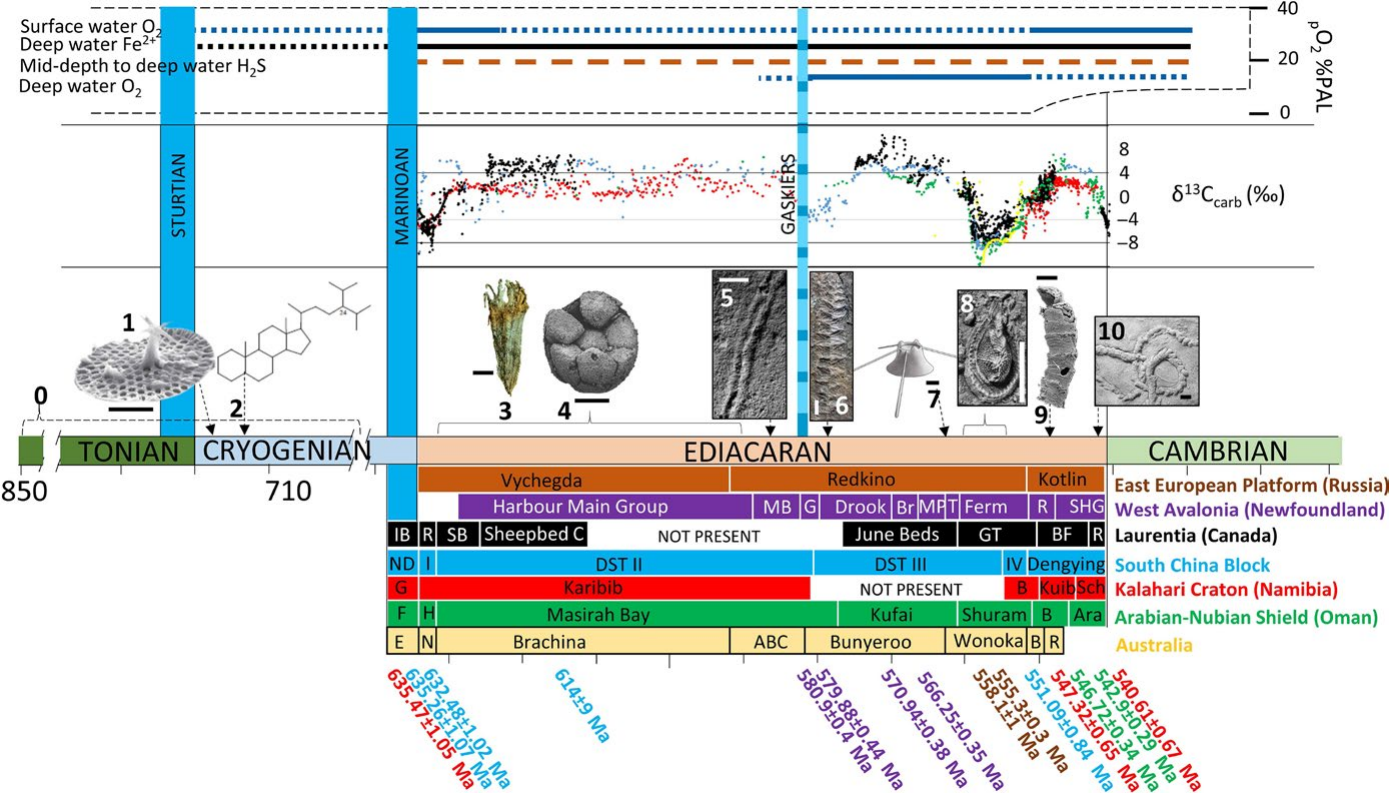
Summary of major changes in ocean chemistry and key biotic events across the Ediacaran‐Cambrian transition. (a) Right: maximum and minimum limits of global ocean‐atmosphere oxygenation (PAL) (Sperling, Wolock, et al., 2015), left: schematic diagram of ocean redox stratification modified after Canfield et al. (2008). (b) Ediacaran C‐isotope compilation modified after Macdonald et al. (2013), and references therein (data and references included in supplementary information). (c) First appearance of major evolutionary milestones: 0, Origin of metazoans from molecular clock dating 850–650 Ma (dos Reis et al., 2015), 1, 812–717 Ma, Phosphatic biomineralisation in microfossils, 15 mile Group, Yukon, Canada (image: Figure 1a SEM image of *Characodictyon*, scale bar = 2 μm; Cohen et al., 2011); 2, 713 Ma, Maximum age of demosponge biomarkers (24‐isopropylcholestane), Huqf Supergroup, Oman (line structure of C skeleton for 24‐ipc; Love et al., 2009); 3, 635–590 Ma, Possible stem‐group Cnidaria *Lantianella laevis,* preserved in black shale of Lantian member II, Anhui Province, South China, scale bar = 3 mm (Yuan et al., 2011); 4, Phosphatised proposed animal embryos of the Doushantuo members II and III at Weng'an section, Guizhou province, scale bar 200 μm (Xiao et al., 1998); 5, Earliest trace fossil evidence for bilaterian motility, Tacuarí Formation, Uruguay, scale bar 2.5 mm (>585 ± 3.3 Ma) (Pecoits et al., 2012); 6, Mistaken Point biota, the rangeomorph *Fractofusus misrai*, Drook Formation (>578.8 ± 1 Ma), scale bar 20 mm (Liu et al., 2015); 7, Sponge spicules and “Multi‐element” metazoan, *Coronacollina acula*, Ediacara Member, Australia scale bar = ~5 mm (equivalent to White Sea assemblage, undated, inferred ~560–550 Ma) (Clites, Droser, & Gehling, 2012); 8, ~558–555 Ma, Motile bilaterian organism *Kimberella quadrata*, scale bar = 10 mm (Fedonkin et al., 2007; Martin et al., 2000); 9, ~550 Ma, Biomineralising reef‐building metazoan, *Cloudina* (Penny et al., 2014), also with predatory borings from the Dengying Formation, South China, scale bar = 200 μm (Bengtson & Zhao, 1992); 10, Ichnofossil *Treptichnus pedum* marks the Ediacaran/Cambrian boundary at the GSSP at Fortunehead, Newfoundland. Example specimen from the Nama Group, scale bar = 10 mm (Wilson et al., 2012). (d) Approximate stratigraphic correlation of major Ediacaran sections which contributing C‐isotope data, modified after Macdonald et al. (2013). East European Platform (EEP) correlation based on biostratigraphy and observations noted by Grazhdankin et al. (2011). Two ash beds within Redkino Formation (Grazhdankin, 2003 and Martin et al., 2000). Avalon ages reported in Pu et al. (2016). Ages recalculated in Schmitz (2012): Oman: Upper ages originally from Bowring et al. (2007). Nama: Ages originally from Grotzinger et al. (1995) and Hoffmann, Condon, Bowring, and Crowley (2004). China: Condon et al. (2005); Liu, Yin, Gao, Tang, and Chen (2009) [Colour figure can be viewed at wileyonlinelibrary.com]

Geochemical investigations of Neoproterozoic sedimentary rocks have revealed a marine landscape characterised by dynamic redox stratification and dramatic, long‐lived perturbations to the carbon isotope record, which accompanied the emergence and early diversification of animals (Figure 1). Molecular clock dating places the origin of crown group Metazoa at 850–650 million years ago (Ma) within the late Tonian to Cryogenian Period (dos Reis et al., 2015; Shields‐Zhou, Porter, & Halverson, 2016). Despite the difficulty in estimating molecular divergence times and the patchiness of the fossil record, this date is in broad agreement with the earliest evidence for Metazoa, as interpreted from demosponge sterols at ~713 Ma (Love et al., 2009). Beyond these biomarker traces, the archive of animal life remains absent until the appearance of credible animal fossils in the Ediacaran Period (635–541 Ma), which reveals diverse ecosystems preserved via a number of taphonomic pathways (Liu, 2016; Narbonne, 2005).

Modern marine environments show differing ecological distributions which correlate with local water column dissolved oxygen concentration. Furthermore, controls on local redox conditions include the degree of productivity as well as the influence of local hydrodynamics. This review considers controls on the redox of Ediacaran marine settings which hosted the earliest metazoan communities. We compile and review published local‐scale redox proxy data from 44 fossiliferous sections, corresponding to six distinct palaeogeographic provinces of the Ediacaran to early Cambrian, in order to explore the physiochemical controls on local redox conditions. Summarised biotic occurrence, palaeolatitude, redox and degree of local basin restriction from the global ocean are given in Table 1. We combine these records with proposed metazoan and complex multicellular eukaryote distribution and ecology. Previously proposed mechanisms for redox variation within each environment are reviewed and discussed in an attempt to clarify the hydrographic controls on local environmental oxygenation.

**Table 1:** Summary of the six palaeogeographic provinces considered in this study with inferred palaeolatitude, degree of restriction, associated biota and dominant redox environment during biotic colonisation

Province	Approximate palaeolatitude (Li et al., 2013)	Environment and connectivity to global ocean	Key biota	Dominant redox environment of habitation
Yangtze Block (South China)	635 Ma: 30–60°N 580 Ma: ~0°N 540 Ma: 0–30°N	Variable connectivity; semi‐restricted intrashelf basins and unrestricted slope‐basin environment. Deep siliciclastic facies within intracontinental basins (e.g., Yangtze Gorges), shallow carbonate facies of elevated margins, and deep siliciclastic facies of the open slope to basin	Early Cambrian Metazoa: articulated sponges, arthropods, motile bilaterian trace‐makers, small shelly fossils (SSFs) and stem lophotrochozoans	Anoxic ferruginous, euxinic and impersistent oxia nearing 520 Ma
			Tubular soft‐bodied and biomineralising metazoans including *Conotubus*,* Cloudina*,* Sinotubulites*, and *Wutubus*. Ediacara‐type fossils including *Yangtziramulus*,* Pteridinium*,* Rangea*, and *Charniodiscus* (Dengying Formation)	Anoxic ferruginous, dysoxic to oxic (inferred from Ce/Ce* data). Impersistent euxinia of equivalent deep Liuchapo Formation
			Doushantuo member IV: Miaohe biota: includes the probable metazoan *Eoandromeda*	Ferruginous
			Doushantuo phosphatised animal embryos and acritarchs Lantian biota: Algae and possible Cnidaria	Ferruginous with euxinia of the open deep slope to basin
Laurentia (North America)	580 Ma: 30–75°S 540 Ma: 10–50°S	Siliciclastic lower slope to basin, shallowing up‐section to mixed carbonate‐siliciclastic. Freely connected rifting to passive margin, equatorial coast of Laurentia	Complex multicellular eukaryotes	Anoxic ferruginous, minor oxic intervals
Kalahari Craton (Namibia)	540 Ma: 30–60°S	Mixed carbonate‐siliciclastic foreland basin fully connected to Brazilides ocean. Two sub‐basins shelf to basin	Soft‐bodied multicellular eukaryotes and biomineralising metazoans, *Cloudina* reefs	Variably anoxic ferruginous, manganous and oxic. Progressive oxygenation?
West Avalonia (Newfoundland)	580 Ma: 30–45°S 540 Ma: 45–60°S	Unrestricted lower slope to basin. Dominantly siliciclastic facies	Complex multicellular eukaryotes	Oxic
East European Platform	580 Ma: ~30°S 540 Ma: 30–60°S	Unrestricted lower slope inferred from drill core. Yskemes‐Vapol’: carbonate dominated. Vychegda‐Kotlin: siliciclastic dominated	Complex multicellular eukaryotes, motile bilaterian metazoans, biomineralising metazoans	Oxic (inferred‐ no direct fossil occurrence in drill core section)
Rio de la Plata Craton (Uruguay)	540 Ma: ~60°S	Unrestricted shelf to slope, openly connected to Brazilides Ocean. Mixed carbonate‐siliciclastic	Biomineralising metazoans (*Cloudina*) and acritarchs	Inferred oxic

### 1.1 The record of Ediacaran macrobiota and metazoans

A candidate for the oldest Metazoa is found in successions of the Doushantuo Formation and the equivalent Lantian Formation in South China (635–590 Ma), which host putative phosphatised animal embryos, and possible Cnidaria, respectively (Figure 1) (Van Iten, Leme, Marques, & Simoes, 2013; Wan et al., 2016; Xiao, Zhang, & Knoll, 1998; Yuan, Chen, Xiao, Zhou, & Hua, 2011). Whilst the metazoan affinity of the Lantian biota remains equivocal, the first appearance of an exceptionally preserved suite of body fossils, which include forms with probable diploblastic and, in some cases, even triploblastic organisation—the Ediacara biota—has been recorded from deep marine siliciclastic strata which bordered the volcanic island arc of Avalonia (~579–575 Ma) (Liu, Kenchington, & Mitchell, 2015). The Ediacara biota are subsequently observed in marine sediments on a global scale, until the Precambrian/Cambrian boundary.

The distinctive fossils in 580–540 Ma stratigraphy have classically been grouped into the Avalon, White Sea and Nama taxanomic assemblages, based on biogeographic and biostratigraphic subdivision (Waggoner, 2003). The Avalon assemblage is the oldest, with fossils noted from a number of marine siliciclastic successions, including sections from Newfoundland (Liu et al., 2015; Narbonne, 2005), Charnwood Forest in England (Wilby, Carney, & Howe, 2011) and the Mackenzie Mountains, Canada (Narbonne, Laflamme, Trusler, Dalrymple, & Greentree, 2014). Examples of Avalon assemblage biota include soft‐bodied rangeomorphs and frond‐like arboreomorphs with isolated occurrences of sponges and triradialomorphs (Laflamme, Darroch, Tweedt, Peterson, & Erwin, 2013). In addition to these forms, the subsequent White Sea assemblage contains the earliest examples of dickinsoniomorphs, erniettamorphs, tetraradialomorphs, pentaradialomorphs, bilateralomorphs, kimberellomorphs and *Eoandromeda* within sections of Siberia (Grazhdankin, 2014), western Russia (Fedonkin, Simonetta, & Ivantsov, 2007), Australia (Gehling & Droser, 2009) and the Yangtze Block, South China (Yuan et al., 2011; Zhu, Gehling, Xiao, Zhao, & Droser, 2008). Examples of the Nama assemblage are represented in successions of the Nama Group, Namibia (Narbonne, Saylor, & Grotzinger, 1997), Dengying Formation, China (Chen et al., 2014), Erga and Chernokamen Formations, Russia, Khatyspyt Formation, Siberia (Grazhdankin, 2014), Miette Group, British Columbia (Hofmann & Mountjoy, 2001) and Wood Canyon, California (Corsetti & Hagadorn, 2000). It has been statistically shown that the Nama assemblage constitutes the assemblage of lowest diversity and contains examples of rangeomorphs, erniettamorphs, arboreomorphs and sponges (Darroch et al., 2015). Recent reconsideration of the assemblage hypothesis supports classic partitioning based on taxonomically distinct groups. However, significant proportions of the palaeogeographically disparate Avalon and White Sea assemblages are seen to have occupied time‐equivalent environments (Boag, Darroch, & Laflamme, 2016).

The earliest bilaterian trace fossil occurrence is observed in middle Ediacaran (>585 ± 3.3 Ma) strata of the Tacuarí Formation, Uruguay (Pecoits et al., 2012). Subsequent deposits of the White Sea area (Russia) and Ediacara member (South Australia) reveal trace fossil evidence for motility alongside co‐preservation of the culprit molluscan trace maker, *Kimberella quadrata* (Fedonkin et al., 2007; Gehling, Runnegar, & Droser, 2014; Martin et al., 2000).

The Ediacaran Period also witnessed the advent of biomineralisation in putative invertebrates. Whilst the genomic toolkit required for this evolutionary innovation was available to microorganisms as early as 812–717 Ma (Figure 1c) (Cohen, Schopf, Butterfield, Kudryavtsev, & MacDonald, 2011), the first skeletal metazoans appear in the fossil record at ~550 Ma (Grant, 1990). The early record of biomineralisation in multicellular organisms is represented in the fossil record by sessile, benthic forms including *Cloudina* (Grant, 1990), the possible lophophorate *Namacalathus hermanastes* (Zhuravlev, Wood, & Penny, 2015), *Sinotubulites* (Chen, Bengtson, Zhou, Hua, & Yue, 2008) and possible sponge *Namapoikia* (Wood, Grotzinger, & Dickson, 2002). The adoption of biomineralisation marks a step‐change in the workings of the global carbon cycle. Colonisation of calcifying biota is seen to have spread throughout shallow and mid‐ramp environments of the terminal Ediacaran, including the Kalahari Craton (Namibia and South Africa), São Francisco Craton (Brazil), Río de la Plata Craton (Uruguay), Yangtze Block (South China), Iberian Peninsula (Spain), Laurentia (Southern Canadian Cordillera and Mexico), the Arabian‐Nubian shield (Oman) and Siberia (Bengtson & Zhao, 1992; Cortijo, Martí Mus, Jensen, & Palacios, 2010; Gaucher & Sprechmann, 1999; Hofmann & Mountjoy, 2001; Hua, Pratt, & Zhang, 2003; Sour‐tovar, Hagadorn, & Huitron‐Rubio, 2007; Warren et al., 2014; Zhuravlev, Linan, Vintaned, Debrenne, & Fedorov, 2012).

The adoption of biomineralisation as a life habit is thought to have required not only the environmental availability of biologically exploitable compounds (e.g., carbonate ions and calcium), but also an external stimulus, with some suggesting the rise of predators as a candidate pressure (Wood, 2011). Earliest evidence for active predation has been documented from organic walled microorganisms within facies of the late Tonian (~780–740 Ma) Chuar Group (Porter, 2016; Shields‐Zhou et al., 2016), whilst suggested predatory borings in *Cloudina* have been reported from the ~550 Ma Dengying Formation, South China (Bengtson & Zhao, 1992), and the Kuibis Subgroup of the Nama Group, Namibia (Brain, 2001).

Of all the organisms represented in the diverse Ediacaran palaeontological record, only a few can be assigned to the Metazoa with any degree of confidence. Examples of the earliest animals include aforementioned biomineralising forms (*Cloudina* etc.), putative sponge fossils (e.g., *Thectardis*; Sperling, Peterson, & Laflamme, 2011), the bilaterian organism *Kimberella* (Martin et al., 2000), the peculiar, octoradially symmetrical *Eoandromeda* (Zhu et al., 2008) and bilaterian organisms responsible for characteristic trace fossils, including terminal Ediacaran *Streptichnus narbonnei* and *Treptichnus pedum* (Jensen & Runnegar, 2005; Wilson et al., 2012). By contrast, the large majority of soft‐bodied organisms represented throughout fossiliferous strata of Ediacaran environments (e.g., rangeomorphs, erniettamorphs, arboreomorphs, etc.) are at present most accurately described as complex multicellular eukaryotes (Laflamme et al., 2013; Liu et al., 2015).

### 1.2 Redox and metazoan ecology

The importance of oxygen provision in enabling high energy yields through aerobic respiration has driven a long‐standing debate on the possibility of an increase in marine dissolved oxygen (beyond a threshold concentration) as a primary factor enabling the rise of animals (Nursall, 1959; Runnegar, 1991; Sperling, Knoll, & Girguis, 2015). Studies on the colonisation and structuring of modern marine ecosystems under variably reducing conditions have shown that well‐oxygenated, nutrient‐rich environments permit sustained habitation by larger organisms, in addition to the potential for biomineralisation (Sperling, Knoll, et al., 2015). Contrastingly benthic metazoan trophic structure in suboxic/anoxic waters is limited to low diversity and is usually characterised by small, unmineralised organisms (Levin et al., 2009; Sperling, Knoll, et al., 2015).

With substantial contention remaining as to the phylogenetic affinity of the majority of soft‐bodied Ediacara biota, it is unclear what environmental requirements may have facilitated their diversification (Liu et al., 2015). In the light of this, hereon the discussion of physiological oxygen requirements within diverse fossil assemblages of the Ediacaran can only be considered to represent end‐member taxa whose presence required elevated levels of dissolved oxygen in the water column, rather than the conditions which dictated establishment of assemblages as a whole.

Recent investigation into one of the most basal modern diploblastic organisms has revealed oxygen concentration requirements between 0.5% and 4% of present atmospheric levels (PAL) (Mills et al., 2014). However, atmospheric oxygen concentrations during the Meso‐Neoproterozoic are poorly constrained and widely debated, with the latest estimates from modelling of proxy data ranging from <0.1% to >4% PAL until at least 800 Ma (Cole et al., 2016; Gilleaudeau et al., 2016; Planavsky et al., 2014; Zhang et al., 2016). By contrast, gas inclusion in ~815 Ma halite from the Officer Basin, South Australia, has been interpreted to suggest an atmospheric oxygen concentration of >10% PAL (Blamey et al., 2016).

Whilst the absolute concentration of atmospheric oxygen is poorly understood, it is clear that the oceans were characterised by continued redox stratification throughout most of the Neoproterozoic, with well‐mixed oxic surface waters being dominantly underlain by anoxic and ferruginous deep waters, and with variable extents of mid‐depth euxinia during certain time periods (Canfield et al., 2008; Guilbaud, Poulton, Butterfield, Zhu, & Shields‐Zhou, 2015; Hood & Wallace, 2015; Johnston et al., 2010; Li, Love, et al., 2012; Sperling, Halverson, Knoll, Macdonald, & Johnston, 2013). Furthermore, considerable lateral heterogeneity likely produced dynamic redox zonation established through patterns of global ocean circulation, localised nutrient recharge and productivity (Reinhard, Planavsky, Olson, Lyons, & Erwin, 2016). Despite such instability, however, marine redox conditions during the Cryogenian Period are thought to have been locally permissive for the evolutionary origin of Metazoa (Sperling, Halverson, et al., 2013).

The geochemical nature of the Ediacaran oceans is characterised by profound and long‐lived fluctuations in the marine carbon and sulphur cycles, which provide further insight into the extent of global ocean‐atmosphere oxygenation. The largest documented negative carbon isotope (δ^13^C) excursion in the geological record, known as the “Shuram‐Wonoka” anomaly (Figure 1b), is recorded in bulk carbonate from palaeogeographically distinct areas, with a disputed initiation at ~575 Ma marking a rapid decline in δ^13^C_carb_ to values as low as −12‰ (Huqf Supergroup, Oman) followed by a slow recovery to positive values by ~553 Ma (Fike, Grotzinger, Pratt, & Summons, 2006). Although the excursion is thought to be globally identifiable, the timing of the onset and recovery to positive δ^13^C_carb_ varies between sections, with a documented duration of between 5 and 50 Myr (Bjerrum & Canfield, 2011; Condon et al., 2005; Jiang, Kaufman, Christie‐Blick, Zhang, & Wu, 2007; Le Guerroué, Allen, & Cozzi, 2006). Although contention remains as to a possible cause (Bjerrum & Canfield, 2011; Burns & Matter, 1993; Cui, Xiao, et al., 2016; Derry, 2010; Grotzinger, Fike, & Fischer, 2011; Kaufman, Corsetti, & Varni, 2007; Knauth & Kennedy, 2009; McFadden et al., 2008; Och & Shields‐Zhou, 2012; Schrag, Higgins, Macdonald, & Johnston, 2013; Swart & Kennedy, 2012), late Ediacaran recovery from the Shuram‐Wonoka excursion has been proposed to represent global oxygenation of the deep ocean (Fike et al., 2006). This is based on evidence supporting enhanced oxidative sulphur cycling (Fike et al., 2006), which was possibly attributable to the advent of sediment ventilation through bioturbation (Wu, Farquhar, & Fike, 2015).

Significantly, however, high‐resolution investigations of local‐scale redox within fossiliferous terminal Ediacaran successions indicate continued dynamism between deposition under anoxic and oxic water column conditions in both deep marine and shallow shelf settings (Och et al., 2015; Sperling, Carbone, et al., 2015; Tostevin, Wood, et al., 2016; Wood et al., 2015). Whilst low atmospheric oxygen concentration in the Neoproterozoic may have been a principal reason for sustained local deep‐water oxygen deficiency, evidence for substantial and long‐standing spatial marine redox heterogeneity within approximately time‐equivalent Ediacaran sections demands consideration of more subtle physical mechanisms.

Many now consider that successful early ecosystems required stable O_2_ above a threshold concentration that was maintained for an ecologically significant timescale (Johnston et al., 2012, 2013; Wood et al., 2015). Under this hypothesis, it was local redox instability which may have delayed proliferation of early animal ecosystems and resulted in the paucity of fossil Metazoa until the late Ediacaran. In summary, the advent of animals is thought to represent the culmination of inherent prerequisite genomic development, physical and chemical change in the marine environment and predation, which together helped drive the evolutionary step towards skeletonisation.

## 2 Controls on Local Redox in Modern and Ancient Environments

Global atmospheric oxygen concentration is ultimately controlled by the balance between oxygen supply via photosynthetic primary production and long‐term burial of reduced elements (e.g., pyrite iron and organic carbon), and oxygen consumption resulting from oxidative weathering of reduced elements within continental sediments and oxidation of reduced gases delivered through volcanic emissions (Canfield, 2014). The primary mechanisms responsible for subsequent atmospheric oxygen delivery to the global ocean include diffusion and efficient physical ventilation of surface waters due to wave agitation and dispersive mesoscale eddies, oxic riverine influx to coastal waters and downwelling of oxygenated surficial water masses (Algeo & Lyons, 2006; Broecker, 1997; Kershaw, 2015; Petsch, 2003). Finally, the concentration and spatial distribution of dissolved oxygen in the marine environment may fluctuate depending on local circulation and the rate and extent of primary production and remineralisation (Helly & Levin, 2004; Petsch, 2003).

### 2.1 Productivity and the biological pump

Marine redox on a local scale is subject to substantial variation resulting from the balance between oxygen supply and biological oxygen consumption through energy‐yielding organic matter oxidation (remineralisation). The initial concentration of organic matter production is primarily dictated by nutrient availability in the surface ocean (Li, Meng, Algeo, & ShuCheng, 2015). Organic matter remineralisation follows a predictable electron acceptor utilisation pathway dictated by the associated energy yield per mole of organic carbon derived from each oxidation reaction, with the highest energy yield achieved through oxidation of free O_2_ during aerobic respiration (Canfield & Thamdrup, 2009). As dissolved O_2_ concentration decreases with organic matter sinking, the preferred electron acceptor first becomes nitrate within the nitrogenous zone followed by a manganous‐ferruginous layer with reduction of continentally derived iron and manganese oxides (Canfield & Thamdrup, 2009; Cheng et al., 2016; Li, Meng, et al., 2015). The underlying euxinic zone is defined by sulphate reduction and build‐up of H_2_S_aq_. The presence or absence of euxinia in an anoxic ocean is partly controlled by the relative fluxes of highly reactive Fe minerals and sulphate (Poulton & Canfield, 2011), in addition to the efficiency of organic carbon delivery from productive surface waters. Furthermore, it has been suggested that long‐standing euxinia demands nitrate depletion as a consequence of the higher free energy yield associated with denitrification over dissimilatory sulphate reduction, and new production must accordingly be sustained via nitrate provision in addition to anoxic N_2_‐fixation (Boyle et al., 2013; Canfield, 2006). In this way, excess bioavailable nitrogen is able to support organic matter production required for oxidation during sulphate reduction, after quantitative denitrification.

Recent model investigations of the biological pump suggest that enhanced efficiency of surface water organic matter oxidation through reduced rates of sinking and/or enhanced rates of respiration is able to effectively lift the oxycline to shallower depths (Meyer, Ridgwell, & Payne, 2016). This supports previous studies invoking oxycline deepening resulting from faster sinking of organic matter due to the consequent increase in remineralisation depth (Butterfield, 2009; Lenton, Boyle, Poulton, Shields‐Zhou, & Butterfield, 2014).

### 2.2 Upwelling and downwelling in the open ocean

Superimposed upon biochemical processes, which locally act to consume oxygen, are environmental factors which influence dissolved oxygen and nutrient distribution. These include changes in salinity and water temperature, alongside hydrodynamic mechanisms that are subject to local variation as a function of intrinsic factors such as palaeobathymetry, and local water column circulation patterns (Petsch, 2003).

Effective downwelling occurs in areas subject to elevated surface density resulting from high salinity and low temperature. In such areas, the dissolved oxygen concentration of surface waters is elevated due to the effect of higher oxygen saturation solubility at lower seawater temperatures (Petsch, 2003). Downwelling in the modern ocean is therefore principally controlled by palaeolatitude, with lower temperature at higher latitudes promoting the formation of oxygenated deep water (Broecker, 1997; Tomczak & Godfrey, 2001). However, regions prone to fresh water dilution as a consequence of low surface evaporation, such as the modern north Pacific, suffer from less efficient downwelling (Bruce, 1983).

By contrast, persistent upwelling systems ordinarily form in mid‐latitudes as a consequence of equatorial current activity related to displacement of surficial water masses via Ekman transport and equatorial divergence (Fuenzalida, Schneider, Garcés‐Vargas, Bravo, & Lange, 2009). Upwelling may develop seasonally or interannually as an effect of differential wind stress and climatic conditions, respectively (Helly & Levin, 2004; Paulmier & Ruiz‐Pino, 2009). Nutrient‐rich upwelling waters lead to stimulated surface ocean productivity driving oxygen depletion of subsurface waters through organic matter oxidation and often resulting in shoaling of the oxycline (Fuenzalida et al., 2009).

### 2.3 Redox distribution in modern environments

#### 2.3.1 Restricted environments

Modern marine environments which lack influence from extensive physical mixing by open ocean current activity include the Black Sea and the Cariaco Basin on the Venezuelan continental shelf (Ho et al., 2004). In these settings well‐mixed oxic surface waters transition to anoxic, commonly euxinic (sulphidic) deeper layers due to strong salinity‐induced density gradients and the absence of efficient physical ventilation mechanisms at depth (Algeo & Lyons, 2006). The maintenance of euxinia in restricted environments is a consequence of high sulphate supply from oxidative continental weathering of reduced sulphur species (e.g., pyrite) alongside hydrogen sulphide production via bacterial sulphate reduction (BSR) in the oxygen‐depleted subsurface water column (Algeo & Lyons, 2006). Under these conditions, available water column ferrous iron delivered to the deep anoxic layer through reductive dissolution of ferric oxides is sulphidised and deposited as pyrite.

Drainage basin area of the enclosing landmass and regional precipitation rate influences the concentration of oxic riverine discharge to the restricted basin which may episodically be of sufficient volume to overcome salinity‐induced stratification and result in short‐term lowering of the oxycline at basin margins (Kershaw, 2015). Furthermore, the relative stability of chemical stratification in restricted basins is a function of the rate of deep‐water renewal related to the degree of basin connectivity with the open ocean as indicated by variations in chemocline depth and depth of the basin margin sill relative to total basin depth (Algeo & Lyons, 2006; Konovalov, Murray, Luther, & Tebo, 2006). More effective restriction and reduced mixing of deep basin waters are implied by lower chemocline and sill depth ratios, respectively (Algeo & Lyons, 2006).

Mechanisms for deep‐water oxygenation include extensive cooling above the oxycline and convective overturn of the stratified basin due to density inversion, in addition to submarine mass wasting brought on by slope instability (Anderson & Devol, 1973; Kershaw, 2015). Additionally, enhanced basin connectivity through eustatic sea‐level rise may result in overflow and breach of well‐mixed, higher density oxygenated waters into the underlying anoxic zone (Konovalov et al., 2006). However, this hyperpycnal incursion may be accompanied by nutrient replenishment and contrastingly result in consumption of oxygen through short‐term elevated organic carbon production and remineralisation (Li, Taylor, Astor, Varela, & Scranton, 2012). The efficiency of these mechanisms towards effective oxygenation of the subsurface is dependent upon their frequency and magnitude with respect to basin volume (Algeo & Lyons, 2006).

#### 2.3.2 Unrestricted continental shelf

Open ocean shelf settings lack bathymetric restriction from the well‐mixed ocean and as a result may be locally subject to vertical and lateral mixing through ocean current activity at mid‐depths, Ekman transport and baroclinic transport of surface waters (Fuenzalida et al., 2009). Some shelf and continental slope areas experience oxygen depletion through local enhancement of the biological pump stimulated by upwelling of nutrient‐rich bottom water (Fuenzalida et al., 2009; Helly & Levin, 2004). This differs from restriction‐induced maintenance of subsurface anoxia, in that oxygen minimum zones (OMZs) on the open shelf exhibit variation in the vertical and lateral positioning of upper and lower boundaries, which are generally maintained through relatively sluggish local circulation (Fuenzalida et al., 2009; Helly & Levin, 2004). Volumetric changes in oxygen minima along continental margins of the modern ocean occur on glacial‐interglacial timescales and are thus identifiable through palaeoredox proxy methods.

Four major OMZ settings in the modern ocean, with dissolved oxygen concentrations <20 μmol/kg, include the eastern south Pacific, the eastern tropical and subtropical north Pacific, the Arabian Sea, and the Bay of Bengal in the northern Indian ocean (Helly & Levin, 2004; Paulmier & Ruiz‐Pino, 2009). A further, weaker OMZ (≥20 μmol/kg dissolved O_2_) is related to upwelling of the Benguela current and occurs off the coast of Walvis Bay, Namibia, in the eastern tropical south Atlantic (Helly & Levin, 2004).

Differential thickness and spatial extent of the OMZ off the coast of Peru in the south‐eastern tropical Pacific correlates well with nutrient input via Ekman‐induced upwelling, whilst the positional offset of the OMZ is an effect of the disconnect between the physical process of upwelling and regional migration of biological activity (Fuenzalida et al., 2009). Transient adjustment of the mixed layer depth along continental margins such as the Namibian shelf and Arabian Sea is induced through strong seasonal differences in wind stress and sea surface temperature, resulting in shoaling of oxygen‐depleted water from depth, in addition to convective mixing (Algeo & Lyons, 2006; Kumar & Narvekar, 2005). Variations in areal extent, thickness and intensity of an OMZ may occur on interannual timescales associated with cyclic changes in sea surface temperature and circulation. An example of this in the modern ocean is the El Niño Southern Oscillation, which is responsible for shrinking the OMZ in the eastern tropical south Pacific during periods of enhanced surface warming (Fuenzalida et al., 2009; Helly & Levin, 2004).

## 3 Recording Palaeoredox in Marine Settings

### 3.1 Global ocean proxies

Oceanic residence times greatly in excess of the rate of global ocean mixing allow a number of redox sensitive trace elements (RSE) to display globally homogeneous open ocean concentrations. Examples include molybdenum, uranium and vanadium, which are commonly enriched in sediments deposited beneath locally anoxic, particularly euxinic, bottom waters. Redox sensitive element enrichments provide a window for interpretation of the global ocean elemental inventory provided that the extent of enrichment has not been impacted by protracted episodes of limited deep‐water renewal brought about by local basin restriction (Algeo & Lyons, 2006; Sahoo et al., 2012, 2016; Scott & Lyons, 2012). In this way, extreme enrichments of RSE within organic‐rich shales are indicative of local euxinia, whilst maximum values may aid interpretation of the global seawater elemental inventory and thus the degree of global marine anoxia (Kendall et al., 2015; Sahoo et al., 2012; Scott & Lyons, 2012; Tribovillard, Algeo, Lyons, & Riboulleau, 2006). Additionally, a number of elements display redox associated isotopic fractionation, of which the most commonly utilised in palaeoenvironmental studies are Mo and U (Andersen et al., 2014; Kendall et al., 2015; Neubert, Nagler, & Bottcher, 2008; Siebert, Nagler, von Blankenburg, & Kramers, 2003; Stirling, Andersen, Warthmann, & Halliday, 2015). Studies of redox sensitive element enrichment and isotopic fractionation continue to aid interpretation of local and particularly global ocean palaeoredox conditions.

### 3.2 Local/regional proxies

#### 3.2.1 Iron speciation

Iron speciation via the technique developed by Poulton and Canfield (2005) allows for localised redox reconstruction through evaluation of the concentration of iron phases considered highly reactive (Fe_HR_) to biological/abiological reduction under anoxic conditions, relative to total iron (Fe_T_). Under oxic conditions, soluble Fe^2+^ is almost entirely oxidised to insoluble Fe^3+^, whilst anoxic conditions can allow transport of Fe^2+^ until water column precipitation is induced (Raiswell & Canfield, 1998). Water column Fe^2+^ may precipitate as pyrite when transported to euxinic settings or may be precipitated as a range of non‐sulphidised minerals (including Fe carbonates and oxides) under anoxic, non‐sulphidic (ferruginous) conditions (Poulton, Fralick, & Canfield, 2004). This augments the detrital influx of Fe_HR,_ potentially giving enrichments in the deposited sediment. The technique of Poulton and Canfield (2005) subdivides these minerals into operationally defined phases, including iron carbonates (e.g., ankerite and siderite), ferric oxyhydroxides (e.g., goethite, lepidocrocite, ferrihydrite and haematite), magnetite and sulphide‐associated iron phases (e.g., pyrite and mackinawite). The sum of Fe_HR_ plus iron bound in poorly reactive or unreactive silicates (geochemically inert on early diagenetic timescales) encompasses the total iron (Fe_T_) content of modern sediments and ancient marine shales (Raiswell & Canfield, 1996, 1998).

Sediments deposited under oxic water column conditions record suppressed Fe_HR_/Fe_T_ (commonly below 0.22) due to the lack of highly reactive iron accumulation in the water column, whereas under anoxic water column conditions, ratios of Fe_HR_/Fe_T_ are typically elevated above 0.38 (Poulton & Canfield, 2005). Where samples have 0.22 < Fe_HR_/Fe_T_ > 0.38, redox interpretation is problematic due to the potential for physical processes such as rapid sedimentation to reduce the rate of Fe_HR_ enrichment under anoxic depositional conditions (Lyons & Severmann, 2006; Poulton & Canfield, 2011). Furthermore, alteration of Fe_HR_ to unreactive iron (Fe_U_) may result in reduced Fe_HR_/Fe_T_ and false oxic interpretation (Poulton and Raiswell, 2002; Raiswell et al., 2008). In these cases, additional consideration of Fe_T_/Al ratios (see below) and poorly reactive Fe contents may allow oxic and anoxic samples to be distinguished (see Cumming, Poulton, Rooney, & Selby, 2013; Poulton, Fralick, & Canfield, 2010).

The iron speciation proxy has the additional advantage of being able to distinguish between euxinic and ferruginous conditions. Under euxinic conditions, the build‐up of water column hydrogen sulphide (H_2_S_aq_) results in sulphidation of iron oxides and formation of iron pyrite (FeS_2_; Fe_py_), leading to elevated Fe_py_/Fe_HR_ (Poulton et al., 2004). Enrichments in Fe_HR_ with low Fe_py_ are considered indicative of ferruginous anoxia (Poulton & Canfield, 2005). Calibration of modern and ancient sediments indicates that where anoxic conditions are inferred by Fe_HR_/Fe_T_ > 0.38, correspondingly elevated ratios of Fe_py_/Fe_HR_ >0.7–0.8 are a strong indicator of euxinic water column conditions, whereas Fe_py_/Fe_HR_ <0.7 are thought to represent ferruginous conditions (März et al., 2008; Poulton & Canfield, 2011; Poulton et al., 2004).

Clarkson, Poulton, Guilbaud, and Wood (2014) enhanced the application of the iron speciation technique via calibration for use on carbonate‐rich sediments, which is of considerable benefit due to the confinement of early calcifiers such as *Cloudina*,* Namacalathus* and *Namapoikia* to carbonate lithologies (Wood, 2011). Clarkson et al. (2014) have shown that the oxic/anoxic Fe_HR_/Fe_T_ thresholds are also valid for carbonates, provided Fe_T_ > 0.5wt%. When Fe_T_ is lower than 0.5 wt%, Fe_HR_/Fe_T_ ratios for oxic samples may show values >0.38 as an artefact of burial dolomitisation and/or diagenetic Fe remobilisation, and thus, Fe speciation should generally be avoided for such samples (Clarkson et al., 2014).

The average ratio of Fe_T_/Al calibrated from studies of Phanerozoic shales and carbonates (0.53 ± 0.11 and 0.55 ± 0.11, respectively) provides additional support when interpreting iron speciation data, with values greater than the upper threshold strongly suggesting local Fe_HR_ enrichment during deposition under a reducing water column (Clarkson et al., 2014; Lyons & Severmann, 2006; Raiswell et al., 2008). Conditions leading to shale Fe_T_/Al values below the calibrated lower threshold are not well understood (Sahoo et al., 2012). However, possible mechanisms for depleted Fe_T_/Al may involve an unusual source material, or the effect of overprinting of accumulated insoluble ferric oxides in oxic shallow facies by resolubilisation and removal during short‐lived shoaling of anoxic water, or depletion of Fe_HR_ by reduction of Fe (oxyhydr)oxide minerals and mobilisation of Fe^2+^ to the water column (the benthic iron shuttle; Lyons & Severmann, 2006; Severmann, Lyons, Anbar, McManus, & Gordon, 2008). The geochemical expression of OMZs with respect to the benthic Fe shuttle shows that enrichment of Fe_HR_ typically occurs at the oxycline beneath the OMZ, whilst sediments within the OMZ commonly show reduced Fe_T_/Al relative to sediments above and below, indicating a possible source of Fe_HR_ for underlying enrichment (Scholz, Severmann, McManus, & Hensen, 2014).

#### 3.2.2 Rare earth elements and cerium anomalies

Distributions of rare earth elements (REEs) within authigenic minerals (e.g., carbonates, phosphates and chert) represent contemporaneous equilibrium between solution complexes and solid phase surface complexes (metal (oxyhydr)oxides, clay and organic matter) provided that there has been no deep‐burial diagenetic modification (McArthur & Walsh, 1984). Cerium is the only REE prone to substantial transformation as a function of ambient seawater E_h_, due to the relatively reduced solubility of oxidised Ce^4+^ and consequent scavenging by Fe‐Mn (oxyhydr)oxides, which leaves the seawater REE pool comparatively depleted in Ce in oxic settings (German & Elderfield, 1990). Characteristic REE profiles with associated anomalous Ce depletion (Ce/Ce*) can therefore be a good indicator of oxia, on condition that there has been no signal modification by later reducing fluids (Bau & Dulski, 1996; Shields, Kimura, Yang, & Gammon, 2004). As such, the entire REE profile must display a distinguishing pattern of diagnostic relative depletions and enrichments indicative of average seawater, from which depletion of Ce relative to the light rare earth elements (LREE: praseodymium to gallium), lanthanum and neodymium indicates likely deposition under oxic water column conditions (Shields et al., 2004; Tostevin, Shields, et al., 2016).

#### 3.2.3 Trace fossils

Studies of modern benthic macrofaunal diversity and complexity under different dissolved oxygen levels imply that traces indicative of motility or active bioturbation, such as *T. pedum*, are restricted to formation by organisms with active metabolic lifestyles that most likely require elevated dissolved oxygen concentrations (Chang, Chronis, Karow, Marletta, & Bargmann, 2006; Wilson et al., 2012). The absence of trace fossil evidence for active motility (Aceñolaza, Germs, & Aceñolaza, 2009), in addition to a lack of evidence for extensive carnivory and predation (Sperling, Knoll, et al., 2015), may support geochemical evidence for widespread anoxic, or low oxygen conditions prior to the late Ediacaran. Indeed, one interpretation for the observed increase in trace fossil evidence nearing the Ediacaran‐Cambrian boundary, and first appearance of *T. pedum*, is a trend towards increasing concentrations or stability of bottom water oxygen (Sperling, Frieder, et al., 2013).

### 3.3 Redox proxy limitations

A shortfall of most redox proxies has traditionally been that they can only be applied to a limited range of lithologies, with most originally calibrated to target fine‐grained siliciclastic sediments such as shale. As discussed above however, recent calibration of iron speciation (Clarkson et al., 2014), alongside redox proxy extraction processes targeting Ce/Ce* (German & Elderfield, 1990; Shields et al., 2004; Tostevin, Shields, et al., 2016) within carbonate‐rich sediments can significantly aid redox interpretation of mixed carbonate‐siliciclastic palaeoenvironments. Differing lithological requirements and proxy sensitivity to different reducing conditions are summarised in Table 2.

**Table 2:** Summary of major palaeoredox proxies, the redox potentials at which they record transformation, and lithologies targeted for their application. (a) Examples of elements which, in addition to providing information on local basin‐scale redox, also enable inference of the nature and extent of global ocean redox and (b) examples of techniques which enable evaluation of basin‐scale redox state

Marine redox proxy	Reduction potential sensitivity	Target lithologies
(a) Global ocean redox		
Examples of redox sensitive elements (RSE)		
V	Sedimentary enrichment begins under E_h_ typical of ${\text{NO}}_{3}^{-}$ reduction	Shale and siltstone
U	Significant sedimentary enrichment occurs rapidly under E_h_ typical of Fe^3+^ reduction	Organic‐rich black shale
Mo	Sequestered by Mn‐Fe oxides under aerobic to mildly reducing conditions. Quantitative drawdown through conversion to particle‐reactive thiomolybdate in the presence of free H_2_S > 11 μmol/L	Organic‐rich black shale
Isotopic fractionation		
δ^238/235^U	Preferential incorporation of ^238^U into insoluble U^4+^ during reduction of U^6+^ and incorporation into organic‐rich mudrocks begins under E_h_ typical of Fe^3+^ reduction	Organic‐rich shale: Elevated δ^238^U under anoxic conditions Carbonate: negligible fractionation during incorporation yielding contemporaneous seawater δ^238^U composition
δ^98/95^Mo	Negligible fractionation during quantitative drawdown of thiomolybdate under highly euxinic conditions. Significant fractionation under weakly euxinic, anoxic non‐sulfidic and oxic conditions	Organic‐rich shales: Represent the proportion of euxinic to less reducing conditions which characterise the global ocean
(b) Local/basin‐wide redox		
REE(Ce/Ce*)	Ce^4+^ scavenged onto Fe and Mn oxides under aerobic to mildly reducing conditions	Phosphorite, chert, carbonate
Fe speciation	Enrichment of Fe_HR_/Fe_T_ allow anoxic conditions to be distinguished from oxic conditions. The degree of sulphidation of Fe_HR_ allows ferruginous and euxinic conditions to be distinguished provided Fe_HR_/Fe_T_ > 0.38	Shale and carbonate

Furthermore, assessing the proportion of the global ocean characterised by euxinia through the use of RSE and isotopic enrichment in shales deposited under locally euxinic conditions suffers the complication that local environments are subject to variation in the degree of euxinia and connectivity to the global ocean. This leads to the necessity for data collection to be accompanied by an evaluation of local water column redox conditions and restriction (e.g., Algeo & Lyons, 2006; Gomes & Hurtgen, 2013; Kendall, Gordon, Poulton, & Anbar, 2011). Studies of modern marine basins subject to variable degrees of restriction from the open ocean indicate that the elemental composition of basinal waters is related to the rate of deep‐water renewal (Algeo & Lyons, 2006; Gilleaudeau & Kah, 2015; Konovalov et al., 2006). A number of studies have explored the potential for the ratio of molybdenum to total organic carbon, and changes in RSE concentration and size of the local seawater sulphate reservoir as geochemical proxies for basin restriction in anoxic sulphidic settings (Algeo & Lyons, 2006; Scott & Lyons, 2012).

Previous studies have also stressed the relative insensitivity of bulk rock techniques to record rapid fluctuations in water column redox conditions, such as those potentially associated with individual fossil occurrences, as samples often represent a significant period of time. Thus, periods of very fleeting oxia and rapid colonisation of substrate by opportunistic biota may be preserved as an overall signature of pervasive anoxia, which in fact may only represent the dominant redox condition during sedimentation of the bulk sample (Johnston et al., 2013; Sperling, Carbone, et al., 2015; Sperling, Knoll, et al., 2015; Wood et al., 2015).

## 4 Ediacaran Redox Synthesis

Apparently conflicting evidence has been published for the extent of open ocean ventilation during the late Neoproterozoic. Molybdenum isotope data have been variously interpreted to indicate widespread ocean oxygenation as early as 700 Ma (Baldwin, Nagler, Greber, Turner, & Kamber, 2013) and substantial water column stratification with continued anoxia at depth up to the early Cambrian (Kurzweil et al., 2015; Wille, Nagler, Lehmann, Schroder, & Kramers, 2008). Whilst iron speciation inherently reflects local/regional redox conditions, extensive compilations from globally distributed shales deposited below wave base can be considered to provide a global redox perspective. Compilations of this style suggest that the majority of the Neoproterozoic (but with important exceptions; see below) was characterised by anoxic ferruginous conditions, which persisted into the Neoproterozoic (Canfield et al., 2008; Guilbaud et al., 2015; Sperling, Wolock, et al., 2015).

### 4.1 Local redox record

To allow direct comparison between sections, iron speciation data have been compiled herein based on calibrated iron phase and major element ratios for depositional conditions. We have employed a conservative framework whereby oxic conditions are indicated by Fe_HR_/Fe_T_ < 0.22, anoxic ferruginous by Fe_HR_/Fe_T_ > 0.38 and Fe_py_/Fe_HR_ < 0.7, and euxinic conditions by Fe_HR_/Fe_T_ > 0.38 and the upper limit of Fe_py_/Fe_HR_ > 0.8. Importantly, where analyses include both siliciclastic and carbonate lithologies, redox variations are shown to be primary and not lithologically determined (Clarkson et al., 2014; Wood et al., 2015). Iron speciation is used herein as a redox proxy baseline, but where available additional proxy data is discussed.

**Figure 2:**
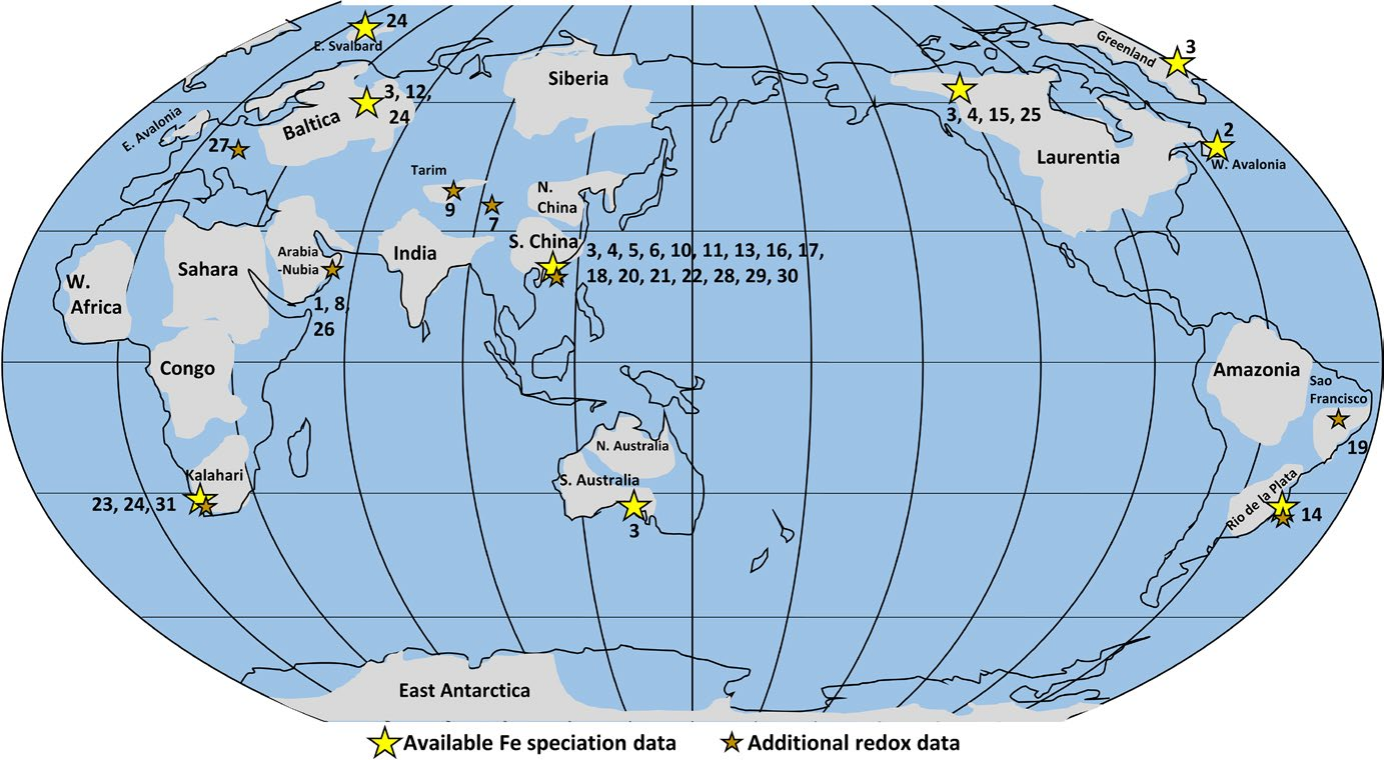
Sections with local palaeoredox proxy data (map modified after Li et al., 2013). Yellow stars indicate available iron speciation data, brown stars indicate available complimentary data of redox (i.e., RSE and REE). Numbers 1–30 relate to major redox studies. 1, Fike et al. (2006); 2, Canfield et al. (2007); 3, Canfield et al. (2008); 4, Shen et al. (2008); 5, Li et al. (2010); 6, Dahl et al. (2010); 7, Shen, Xiao, Zhou, Kaufman, and Yuan (2010); 8, Schroder and Grotzinger (2007); 9, Shen et al. (2011); 10, Sahoo et al. (2012); 11, Wang et al. (2012); 12, Johnston et al. (2012); 13, Och et al. (2013); 14, Frei et al. (2013); 15, Johnston et al. (2013); 16, Fan et al. (2014); 17, Feng et al. (2014); 18, Yuan et al. (2014); 19, Spangenberg, Bagnoud‐Velásquez, Boggiani, and Gaucher (2014); 20, Li, Planavsky, et al. (2015); 21, Kendall et al. (2015); 22, Och et al. (2015); 23, Wood et al. (2015); 24, Sperling, Wolock, et al., 2015; 25, Sperling, Carbone et al. (2015); 26, Osburn, Owens, Bergmann, Lyons, and Grotzinger (2015); 27, Kurzweil et al. (2015); 28, Han and Fan (2015); 29, Jin et al. (2016); 30, Sahoo et al. (2016); 31, Tostevin, Wood, et al., (2016) [Colour figure can be viewed at wileyonlinelibrary.com]

**Figure 3:**
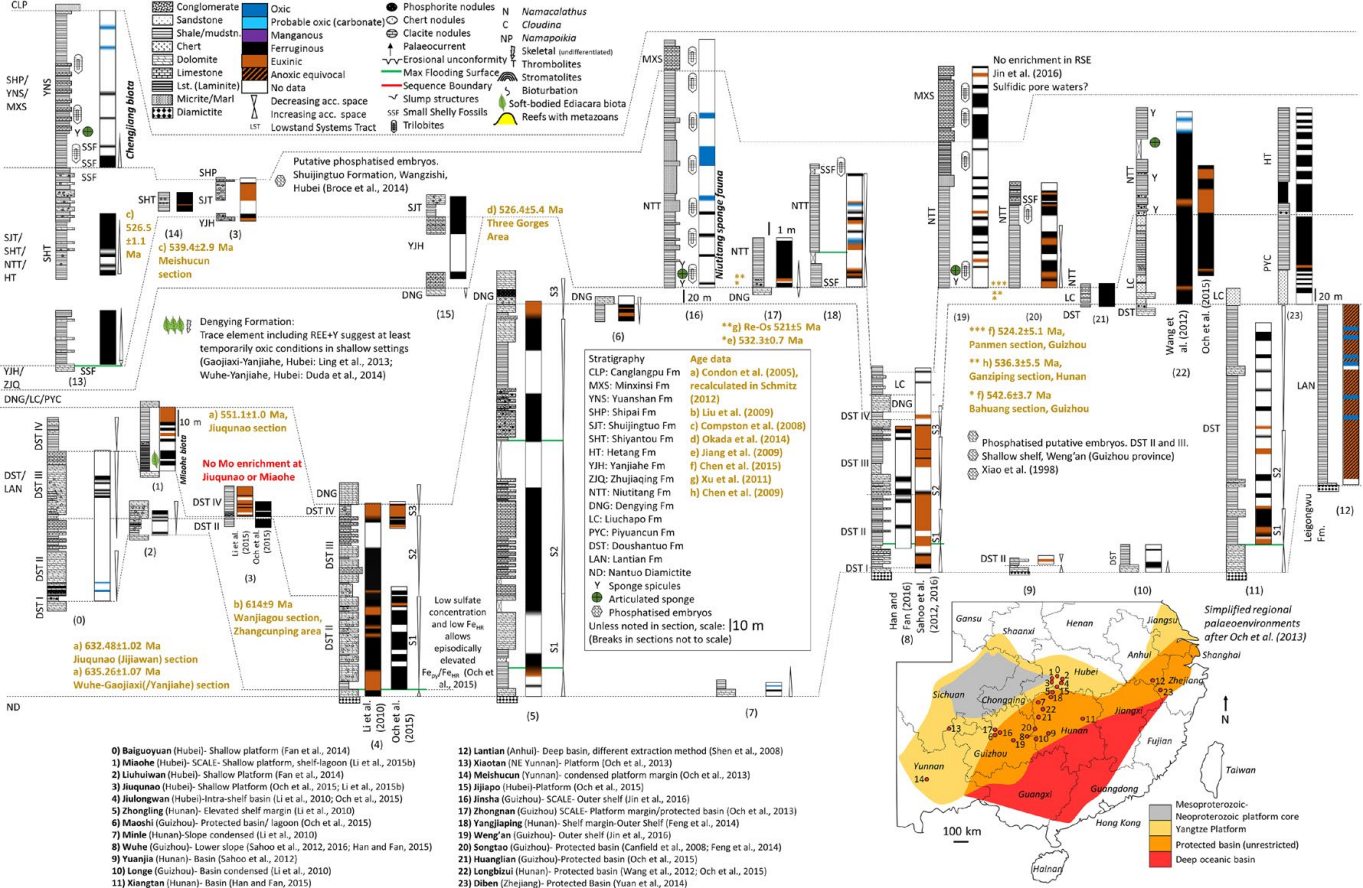
Local redox from variably restricted platform to basin environments of the Yangtze Block, South China (635–520 Ma). See inset for reconstruction of the depositional environments (modified after Och et al., 2013). Schematic depiction of redox by colour. Black: anoxic ferruginous (Fe_HR_/Fe_T_ > 0.38, Fe_py_/Fe_HR_ < 0.7), brown: euxinic (Fe_HR_/Fe_T_ > 0.38, Fe_py_/Fe_HR_ > 0.8), blue: oxic (Fe_HR_/Fe_T_ < 0.22) and white: equivocal (0.22 > Fe_HR_/Fe_T_ < 0.38) [Colour figure can be viewed at wileyonlinelibrary.com]

**Figure 4:**
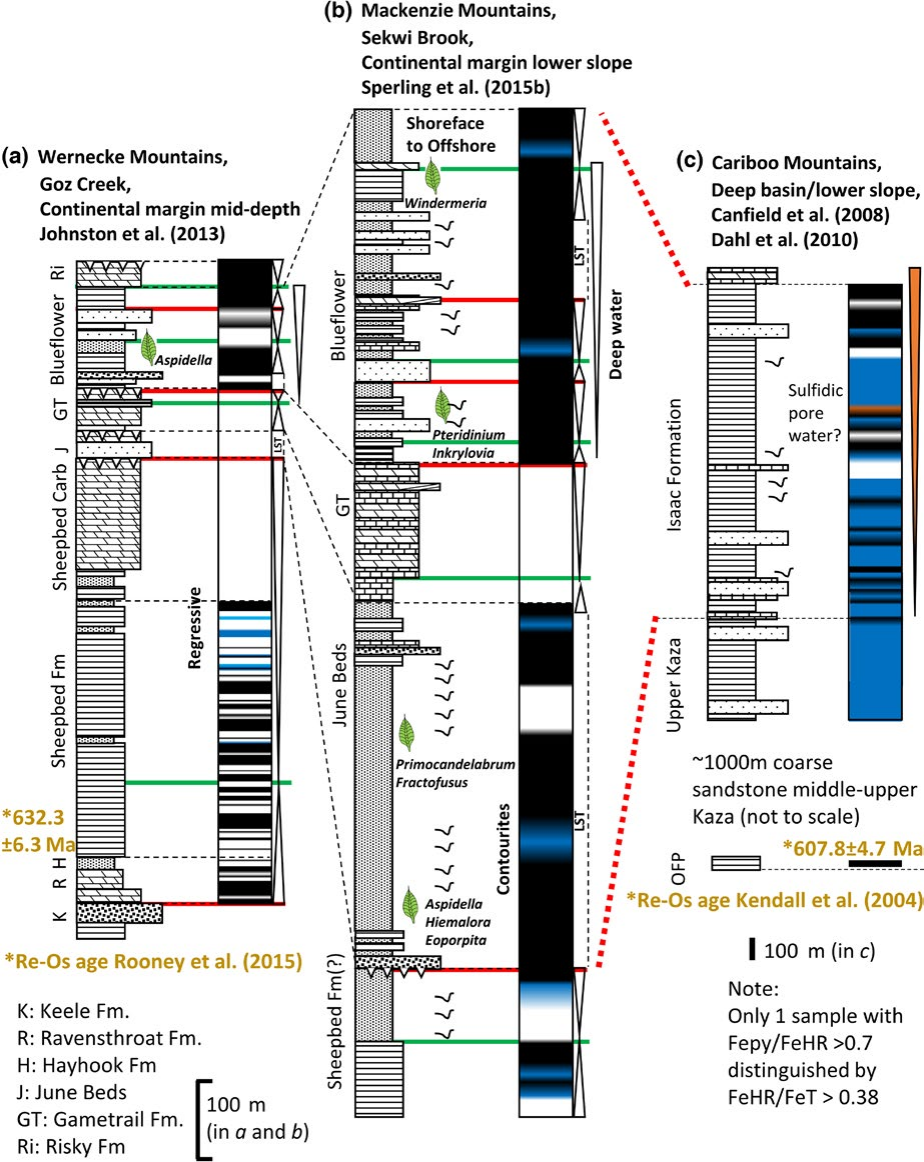
(a–c) Local redox from deep‐water successions of the Windermere Supergroup deposited on northern margin of Laurentia (sections of Yukon Territory, Northwestern Territories and British Columbia Canada) (~632–540 Ma). See Figure 3 for legend. (red dotted lines: tentative temporal correlation after Yonkee et al., 2014) [Colour figure can be viewed at wileyonlinelibrary.com]

**Figure 5:**
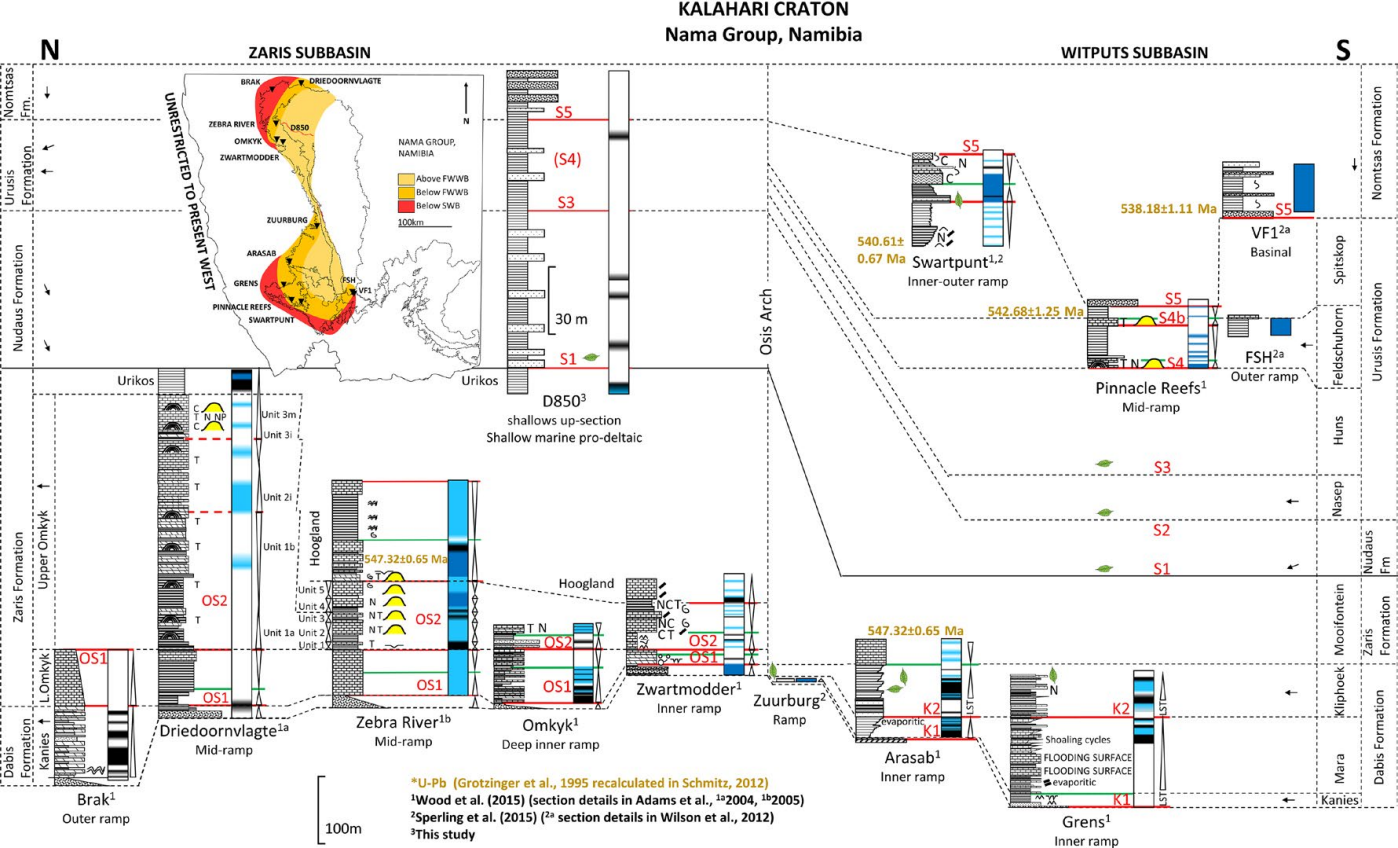
Local redox of the Nama Group deposited in the northern and southern sub‐basins of the Nama foreland basin, Kalahari Craton, Namibia (~550–538 Ma). See Figure 3 for legend [Colour figure can be viewed at wileyonlinelibrary.com]

**Figure 6:**
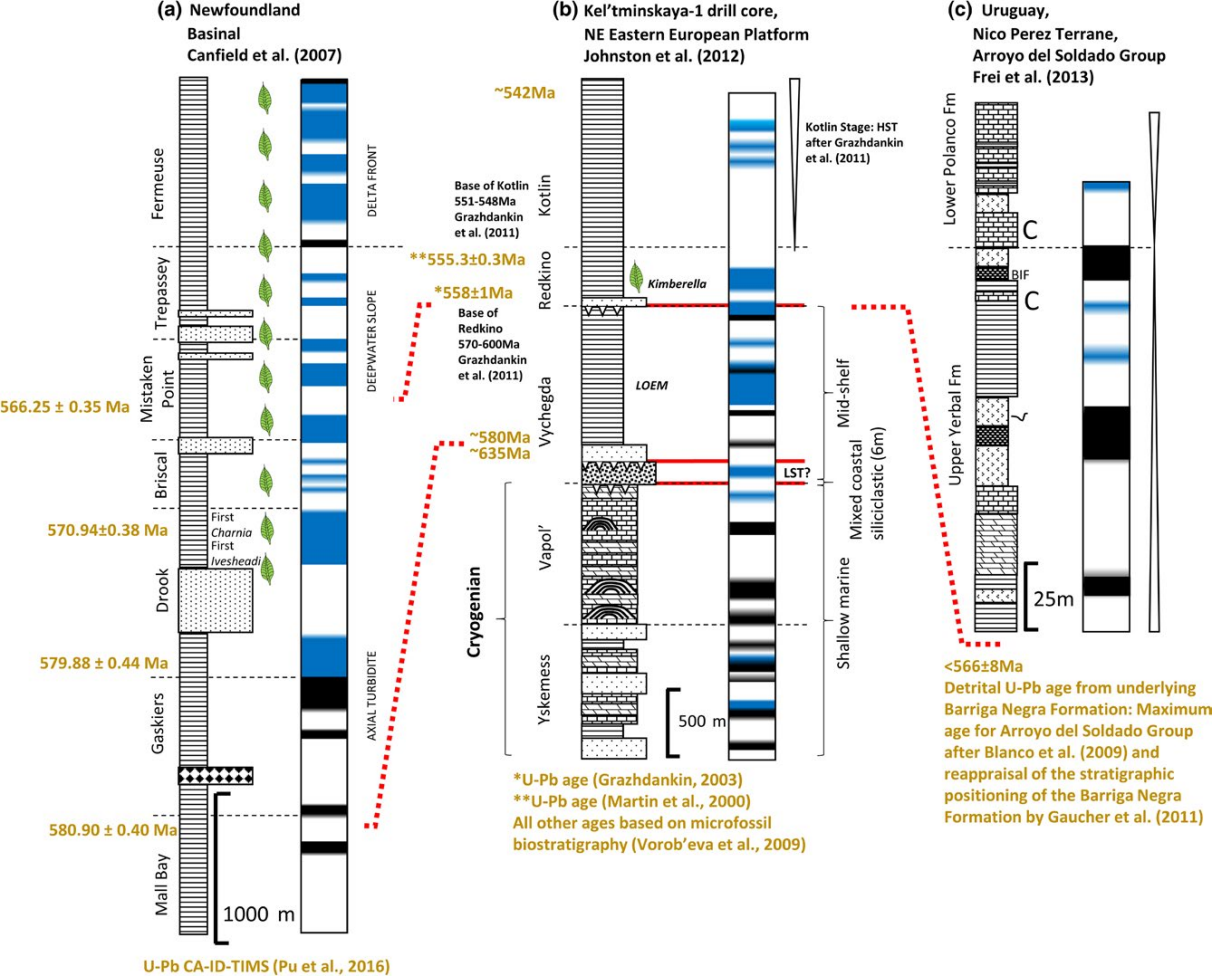
(a–c) Local redox of deep‐water successions deposited in unrestricted lower slope settings of a) Western Avalonia, Newfoundland (~584 Ma to <565 Ma) and (b) the East European Platform (EEP), western Russia (>635 Ma to ~542 Ma) and (c) shallow platform deposits of the Río de la Plata Craton, Uruguay (<566 Ma). Red dotted line: approximate temporal correlation. See Figure 3 for legend [Colour figure can be viewed at wileyonlinelibrary.com]

We consider 44 sections with accompanying Fe speciation data with the aim of reviewing local water column redox within platform to basin environments bordering the Yangtze Block, Laurentia, Kalahari Craton, Avalonia, the East European Platform (EEP) and Río de la Plata Craton (Table 1, Figures 2, 3, 4, 5, 6). Due to the difficulty associated with ascertaining an unambiguous mechanism for exceptionally low Fe_T_/Al, samples which record values below the lower threshold (0.42) are not considered in this collation unless stated specifically in the text. Additional proxy indicators of local redox are also discussed where available and include redox sensitive trace element concentrations (RSE), and REE profiles.

#### 4.1.1 Yangtze block

Richly fossiliferous deposits of the Ediacaran Yangtze Block include the Doushantuo and overlying Dengying formations which contain multiple examples of possible early animals, including aforementioned phosphatised embryos, *Lantianella*,* Eoandromeda* and soft‐bodied and biomineralising tubular forms *Cloudina, Sinotubulites* and *Wutubus,* which are key to deciphering possible environmental requirements of earliest animal ecosystems (Cai, Hua, Schiffbauer, Sun, & Yuan, 2014; Chen et al., 2014; Hua et al., 2003; Van Iten et al., 2013; Zhu et al., 2008).

Intense study of Ediacaran to early Cambrian sections of the Yangtze Block has allowed unparalleled detail in palaeoredox reconstruction across an array of palaeodepth profiles, despite difficulty in coherent determination of lateral equivalence between some formations and members (Figure 3). Basin reconstruction and tentative sequence stratigraphic correlation have been made possible by concerted studies of ash bed dating and detailed δ^13^C chemostratigraphy (Figure 3) (Chen, Wand, Qing, Yan, & Li, 2009; Chen, Zhou, Fu, Wang, & Yan, 2015;Compston, Zhang, Cooper, Ma, & Jenkins, 2008; Condon et al., 2005; Cui et al., 2015; Jiang, Kennedy, Christie‐Blick, Wu, & Zhang, 2006; Jiang, Kaufman, Christie‐Blick, Zhang, & Wu, 2007; Jiang, Shi, Zhang, Wang, & Xiao, 2011; Jiang, Pi, Heubeck, Frimmel, Liu, Deng, Ling, & Yang, 2009; Wang, Jiang, Shi, & Xiao, 2016; Zhu, Zhang, & Yang, 2007; Zhu et al., 2013).

Recent reassessment of stratigraphic equivalence and completeness between studied sections of the Yangtze Block through analysis of sedimentary facies architecture and chemostratigraphic correlation has enabled nuanced understanding of the complex palaeobathymetry which existed during deposition (Cui et al., 2015; Jiang et al., 2011; Vernhet & Reijmer, 2010; Wang et al., 2016; Zhu et al., 2013). Reconstruction reveals a broad shallow platform to the modern northwest with contemporaneous formation of small intrashelf lagoons and restricted deep basins within grabens during initial rifting (e.g., Yangtze Gorges). The intrashelf basin environment was bordered by an elevated shelf margin which transitioned down slope to a large, deep basin (Nanhua basin) to the modern southeast which was connected to the open ocean (Sahoo et al., 2016; Zhu et al., 2007). Abundant rift‐related and block faulted grabens were gradually incorporated into a broad passive continental margin during deposition of Ediacaran sediments, with changing relative sea level enabling affecting restriction of intrashelf basin environments (Jiang et al., 2011; Zhu et al., 2007).

##### Doushantuo Formation (635 to >551 Ma)

Facies of the Doushantuo Formation include shallow peritidal carbonate platform deposits (e.g., Xiaofenghe, Baiguoyuan, Liuhuiwan, Jiuqunao, Miaohe), isolated subtidal shales and carbonates of the intrashelf basin (Jiulongwan sections), mixed carbonate and siliciclastic deposition of the elevated margin rim (e.g., Zhongling) and slope to basinal minor carbonates and shales (e.g., Wuhe, Xiangtan and Lantian) (inset Figure 3) (Cui et al., 2015; Fan et al., 2014; Jiang et al., 2011; Li et al., 2010; Och et al., 2015; Sahoo et al., 2012; Vernhet & Reijmer, 2010; Xiao et al., 2012; Zhou & Xiao, 2007; Zhu et al., 2007). With increasing depth, mixed carbonate‐siliciclastic environments gradually transition to shale‐dominated facies, and Doushantuo stratigraphy is reassigned in the deep basin to the Lantian Formation (Shen, Zhang, & Hoffman, 2008). Deposition of the Doushantuo Formation is constrained by U‐Pb ages of 635.2 ± 0.6 Ma and 551.1 ± 0.7 Ma (Condon et al., 2005; but see An et al., 2015).

In the Yangtze Gorges area, the Doushantuo Formation has classically been subdivided into four lithostratigraphic members which, in ascending order, comprise the thin basal cap dolostone of member I, 80–120 m of shale with occasional medium‐bedded dolostone and chert nodules of member II, 40–60 m of banded and lenticular chert interbeds and dolostone of member III, and a locally absent 10‐m‐thick black shale unit of member IV which commonly exhibits large dolomite concretions (Liu, Yin, Chen, Tang, & Gao, 2013).

Doushantuo members II and III contain a notably diverse suite of large acanthamorphic acritarchs within chert nodules and phosphorites, alongside vase‐shaped microfossils, probable phosphatised animal embryos, multicellular algae and cyanobacteria within semi‐restricted and shallow shelf settings which together comprise the “Weng'an biota” (Liu et al., 2013; McFadden et al., 2008; Xiao, Zhou, Liu, Wang, & Yuan, 2014; Xiao et al., 1998). Controversy surrounding fossilised Weng'an embryos has provoked numerous studies (e.g., Huldtgren et al., 2011); however, recent contributions support an animal affinity as originally proposed (Schiffbauer, Xiao, Sharma, & Wang, 2012; Xiao et al., 1998). Additional extensive study of acritarch taxonomy as a tool for biostratigraphic correlation of the Doushantuo Formation has been made possible through appreciation of three dimensional morphology via acid maceration of well‐preserved specimens entombed within phosphorites at the type section of Weng'an (Guizhou) (Xiao et al., 2014).

Fossiliferous shales of the Lantian Formation member II contain an assemblage of probable in situ multicellular eukaryotes which include *Chuaria circularis*, fan‐shaped macroalgal forms and potential conulariid‐type Metazoa (Van Iten et al., 2013; Wan et al., 2016; Yuan et al., 2011). Recent systematic description of an expanded Lantian fossil sample set has assigned the proposed medusozoan to the morphospecies *Lantianella laevis,* and a further two morphogenera have been assigned to a suite of fossils which share features similarly suggestive of possible stem‐group cnidarian affinity (Van Iten et al., 2013; Wan et al., 2016). In sum, putative animal embryos of Doushantuo members II and III and the “Lantian biota” of deep‐water Lantian member II potentially include examples of the oldest metazoan organisms in the fossil record.

Overlying organic‐rich shales at the base of Doushantuo member IV (≤580 Ma, >551 Ma) contain an assemblage of carbonaceous compressions which likely represent green, and potentially red and brown fossil algae alongside possible Metazoa including the eight‐armed *Eoandromeda octobrachiata* at shallow shelf Miaohe and Weng'an sections and deep‐water deposits at Wenghui (An et al., 2015; Condon et al., 2005; Xiao, Yuan, Steiner, & Knoll, 2002; Zhu et al., 2008). Doushantuo member IV is thus commonly referred to as the Miaohe member after the distinctive “Miaohe biota” assemblage at the type locality (Xiao et al., 2002).

A sequence stratigraphic framework across the shelf and basin has been proposed which dissects Formation deposition during two‐and‐a‐half cycles of accommodation change equating to Stages 1–3, of which regressive Stage 3 spans the Doushantuo‐Dengying boundary (McFadden et al., 2008; Zhu et al., 2013). At Jiulongwan, Stage 1 begins with relative sea‐level rise during deposition of the basal cap dolostone and shales of lower member II, followed by subsequent regression represented by sedimentological indicators of facies shallowing (McFadden et al., 2008). The transgressive surface capping Stage 1 lacks signs of exposure and is immediately overlain by deep‐water facies which show an increase in sedimentological indicators of shallowing during regression throughout Stage 2. Abrupt deepening is re‐initiated at the base of Stage 3 correlating to the transgressive surface between members III and IV (McFadden et al., 2008). This repeated sequence is permitted through long‐term eustatic sea‐level rise across the Ediacaran‐Cambrian boundary (Haq & Schutter, 2008).

Importantly, recent integrated chemostratigraphic investigation of the Doushantuo Formation suggests regionally variable completeness of δ^13^C profiles and infers either truncation in shallow marine settings or a diachronous Doushantuo‐Dengying boundary (Cui et al., 2015). Consequently, it is expected that regional palaeoenvironmental reconstruction based on equivalence across platform to basin sections may be reinterpreted through future chemostratigraphic studies.

**Figure 7:**
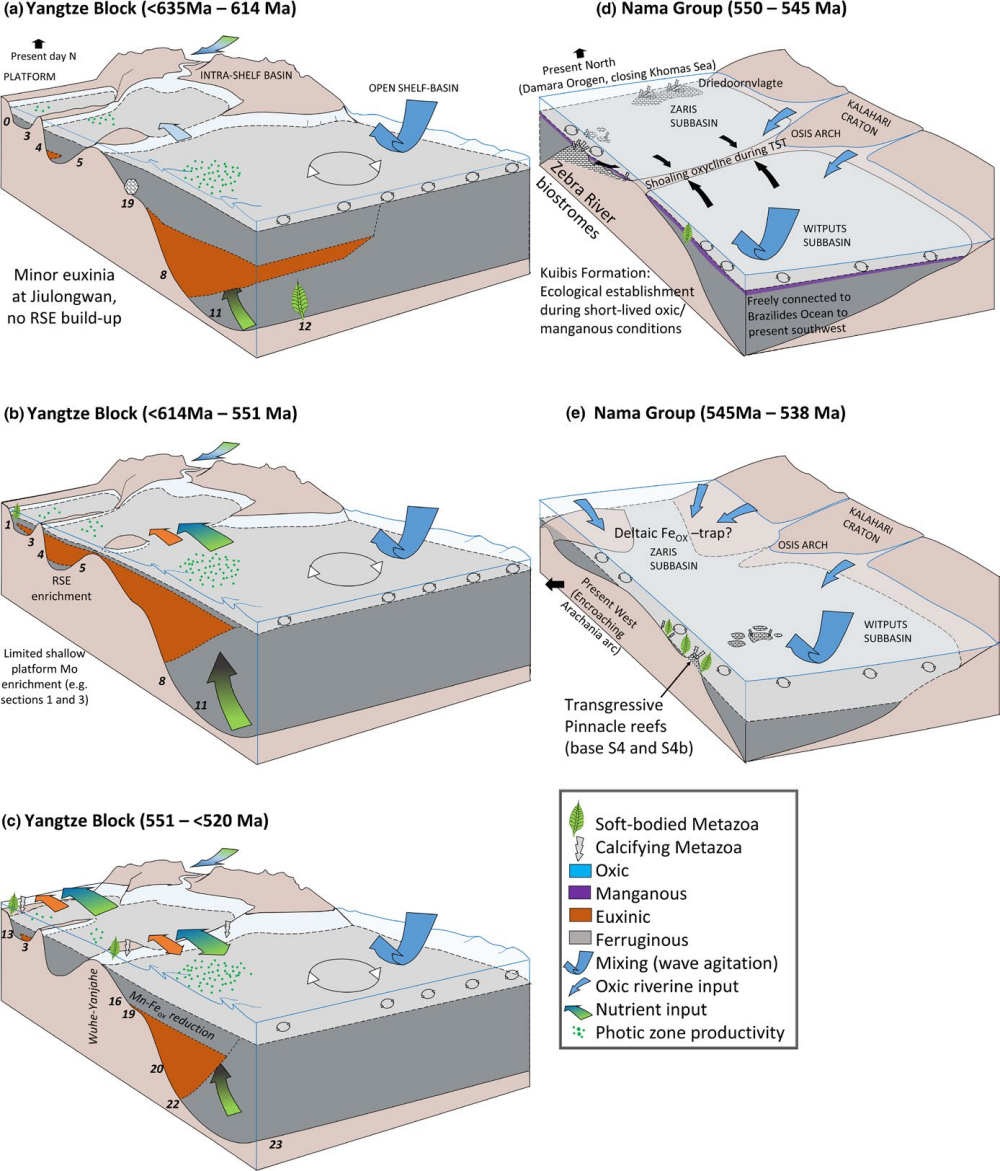
(a–e) Basin‐scale reconstructions of redox conditions for key successions of the Ediacaran‐Cambrian (a–c) Yangtze Block, South China and (d, e) Nama Group, Namibia. A key to section numbers of the Yangtze Block is provided in Figure 3. (a) Doushantuo/Lantian Formations Members I‐III (<635 Ma to 614 Ma): Deep‐water restriction of the Yangtze Gorges intrashelf basin, and euxinia of the unrestricted slope at Wuhe. (b) Miaohe Member (<614 Ma to 551 Ma): Sea‐level rise and reduced restriction of the Yangtze Gorges intrashelf basin. Semi‐restricted conditions are sustained at more proximal sections (e.g., Jiuqunao) evident from continued lack of Mo enrichment (Li, Planavsky, et al., 2015; Och et al., 2015). (c) Dengying to early Cambrian Formations (<551 Ma to <520 Ma): Deposition during continued eustatic sea‐level rise resulted in reduced restriction of proximal intrashelf basins. Whilst euxinia continued to intermittently characterise platform and slope settings, there is some evidence to suggest deepening of the chemocline with first appearance of episodic oxia recorded in deposits of the upper Niutitang Formation at basinal Longbizui. (d) Kuibis Subgroup (550 Ma to <547 Ma): Deposits of the Witputs sub‐basin initially record heterogeneous redox with dominantly anoxic ferruginous conditions followed by subsequent reef growth confined to transgressive systems tract of the Zaris sub‐basin and skeletal metazoan ecology influenced by incursions of anoxia during shoaling of the chemocline. Manganous zone suggested after regional Ce/Ce* study of Tostevin, Wood, et al. (2016). (e) Schwarzrand Subgroup (<547 Ma to 538 Ma): Oxic conditions dominated the Witputs sub‐basin but ferruginous anoxia is documented in the Zaris sub‐basin, suggesting deltaic trapping of Fe oxides and a signature of false anoxia [Colour figure can be viewed at wileyonlinelibrary.com]

The spatial distribution of local redox observed within the Doushantuo Formation (Figure 3) is schematically illustrated in Figure 7a and supports localised development and maintenance of a metastable zone of euxinia on the openly connected lower slope at Wuhe (Figure 3, section 8), which episodically encroached into the deeper basin as supported by intervals of elevated Fe_HR_/Fe_T_ and Fe_py_/Fe_HR_ at Xiangtan (Figure 3, section 11) (Han & Fan, 2015; Sahoo et al., 2016). Intrashelf basins represented at sections such as Jiulongwan (Figure 3, section 4) were only surficially connected to the open ocean and exhibit negligible RSE enrichment and elevated δ^34^S, suggestive of intervals of sulphate depletion brought on by basin restriction (Bristow et al., 2009; Cui et al., 2015; Jiang et al., 2011; Och et al., 2015; Sahoo et al., 2012; Vernhet & Reijmer, 2010; Zhu et al., 2013). Sulphate limitation in a closed system during extended periods of basin restriction or effective disconnect between pore water and seawater will tend to drive the sulphur isotope composition of sedimentary pyrite (δ^34^S_py_) towards heavier values, thus reducing the offset between the isotopic composition of seawater sulphate (preserved in carbonate associated sulphate) and pyrite (Δ^34^S_CAS‐py_) through continued BSR of the increasingly isotopically enriched sulphate reservoir (Gomes & Hurtgen, 2013). Sporadic euxinia inferred from intermittently elevated Fe_py_/Fe_HR_ at Jiulongwan (Li et al., 2010) is thought to be a consequence of episodically low Fe_HR_ supply in the dominantly ferruginous depths of the intrashelf basin during deposition of Doushantuo members II–III (Och et al., 2015). Yet more proximal settings on the shallow platform (e.g., Baiguoyuan; Figure 3, section 0) show evidence for infrequent deposition within well‐mixed, oxic shallow waters which punctuate dominantly ferruginous deposition (Fan et al., 2014).

Variations in thickness of the euxinic zone are thought to be functionally equivalent to spatial variability observed in modern open marine OMZs related to high productivity stimulated through nutrient upwelling (Li et al., 2010; Och et al., 2015; Sahoo et al., 2016). Sustained euxinic conditions in unrestricted settings such as those seen to have characterised the open slope at Wuhe (Han & Fan, 2015; Sahoo et al., 2012, 2016) require both high levels of organic matter supplied by surface water productivity and at least locally elevated influxes of marine sulphate capable of supporting build‐up of H_2_S_aq_, after quantitative pyritisation by available highly reactive iron (Poulton & Canfield, 2011).

Uranium and molybdenum isotope data from euxinic shales of the Yangtze Block have been interpreted to indicate a global increase in oceanic dissolved oxygen concentrations throughout the Ediacaran (Chen, Ling, et al., 2015; Kendall et al., 2015). However, secular organic matter‐normalised RSE enrichment and depletion within euxinic shales of the Doushantuo Formation have been interpreted to support limited global ocean Mo scavenging and temporarily widespread ocean oxygenation (Kendall et al., 2015; Sahoo et al., 2012, 2016; Scott et al., 2008).

A suggested model for the initiation of Mo enrichment and the trend towards more negative δ^34^S_py_ during Doushantuo member IV within the intrashelf basin at Jiulongwan proposes progressive landward incursion of the lower slope euxinic wedge into isolated platform environments under a regime of rising sea level (Figure 7b, Li et al., 2010; Och et al., 2015). The transgressive surface at the Doushantuo member III/IV boundary therefore likely represents an increase in sill depth ratio and basin connectivity. Continued local basin restriction of yet more proximal sections, including Jiuqunao, is thought to account for a corresponding lack of RSE enrichment and elevated δ^34^S_py_ at this time (Och et al., 2015). Additional iron speciation and RSE data collected at Jiuqunao and nearby Miaohe sections corroborate ferruginous anoxia for the lowermost deposits of Doushantuo member IV, but also suggest a trend towards more euxinic conditions within overlying shales (Li, Planavsky, et al., 2015). Importantly, however, inception of euxinic conditions at Miaohe is seen to post‐date fossil occurrence of the Miaohe biota (Li, Planavsky, et al., 2015). Limited Mo enrichment within both Jiuqunao and Miaohe sections (Li, Planavsky, et al., 2015) is consistent with the model of Och et al. (2015) for continued partial restriction of intrashelf sections, and accompanying δ^15^N data at Jiuqunao have been interpreted as evidence for restriction‐induced nitrate limitation which may have precluded the maintenance of euxinia (Och et al., 2015).

Published Ce/Ce* data of the Doushantuo Formation are in broad agreement with iron speciation at Jiulongwan, supporting deposition beneath a redox stratified water column with some evidence for a trend towards more persistent anoxia up‐section (Cui et al., 2015; Ling et al., 2013; Shields et al., 2004; Zhou, Jiang, Xiao, Chen, & Yuan, 2012).

Basinal open ocean deposition at Xiangtan is defined by dominantly ferruginous conditions where organic matter was depleted (Han & Fan, 2015; Li, Meng, et al., 2015). Under this model, nearshore and distal Fe sources are thought to have been distinct, with the shallow manganous‐ferruginous zone permitted through reductive dissolution of detrital Fe and Mn oxides (Li, Meng, et al., 2015). In contrast, anoxic deep waters were typically enriched in soluble reduced Fe^2+^ derived from long‐term hydrothermal build‐up, in addition to reduction of iron oxides and mobilisation of Fe to depth (Li, Meng, et al., 2015; Lyons & Severmann, 2006; Severmann et al., 2008).

A number of samples in the basinal Lantian section indicate low Fe_HR_/Fe_T_, possibly corresponding to sedimentation under oxic water column conditions. However, the extraction procedure used by Shen et al. (2008) at this locality does not isolate carbonate‐bound iron (Johnston et al., 2013; Poulton & Canfield, 2005; Sperling, Carbone, et al., 2015), and given that inferred oxic samples show Fe_HR_/Fe_T_ bordering the upper calibrated threshold for identification of oxic conditions (lowest sample value of 0.19), these data should be treated with caution. Despite methodological issues associated with Fe phase extraction, Fe speciation data of Shen et al. (2008) unambiguously point to a predominantly anoxic water column during Lantian member II. Additional data for pyrite framboid size, RSE, organic carbon, total sulphur, and δ^34^S_py_ of *Chuaria*‐bearing strata have helped constrain deposition of Lantian member II shales under episodically suboxic/oxic conditions (Guan et al., in press). It has therefore been suggested that if the physiology of the Lantian biota demanded less reducing conditions, intervals conducive to habitation may have been very brief (Yuan et al., 2011).

##### Terminal Ediacaran to early Cambrian Formations (551–520 Ma)

Shallowing associated with continued regression during Stage 3 resulted in the deposition of the widespread dolomitic Dengying Formation (551–541 Ma) in shallow and mid‐depths. Down slope, the Dengying Formation is reassigned to interbedded cherts and shales of the lower Liuchapo formation (Wang, Chen, Yan, Wei, & Xiang, 2012). The Dengying, Yanjiahe and Zhujiaqing Formations have been assigned a minimum age of 539.4 ± 2.9 Ma through U‐Pb SHRIMP zircon dating of the middle (Zhongyicun) member deposits (Compston et al., 2008). Corresponding deposition of the deeper water Liuchapo Formation is corroborated by an upper age of 536.3 ± 5.5 Ma (Chen et al., 2009). Based on first appearance of *T. pedum,* the Ediacaran‐Cambrian boundary is conventionally placed at the Daibu/Zhongyicun boundary within the Zhujiaqing Formation (lower Yanjiahe) or deep‐water equivalent Liuchapo/Niutitang boundary (Zhu et al., 2003).

In the Yichang area, the Dengying Formation is subdivided into the lower shallow marine Hamajing dolostone member, middle richly fossiliferous transgressive micritic limestone of the Shibantan member, and upper regressive Baimatuo dolostone member (Duda et al., 2014; Ling et al., 2013). The Dengying Formation preserves an assemblage of Ediacaran soft‐bodied organisms within bituminous limestones of the Shibantan member, including the frond‐like *Paracharnia*,* Rangea*,* Pteridinium*, the enigmatic *Yangtziramulus zhangi*, and the tubular *Wutubus annularis* (Chen et al., 2014). All of these fossils are found in association with abundant trace fossils (e.g., *Lamonte trevallis*), indicating the co‐occurrence of motile bioturbating organisms that are thought to have actively mined nutrients, and potentially oxygen, from contemporaneous microbial mats (Chen et al., 2013; Duda et al., 2014; Meyer et al., 2014). Biomineralising *Sinotubulites* are recorded from the upper Shibantan member and both *Sinotubulites* and *Cloudina* are noted from the Baimatuo member of western Hubei (Chen, Chen, & Qian, 1981; Chen et al., 2008). *Sinotubulites* and *Cloudina* have also been described from Shibantan and Baimatuo‐equivalent shallow platform carbonates of the Gaojiashan and Beiwan members in southern Shaanxi province, where they immediately overlie strata hosting the soft‐bodied tubular organism *Conotubus hemiannulatus* (Cai et al., 2014; Cui, Kaufman, et al., 2016; Hua et al., 2003). To date, no evidence has been presented for reef‐building by *Cloudina* in Dengying Formation carbonates and all specimens appear to occupy a “mat‐sticker” mode of life (Cai et al., 2014). Possible borings have also been described in specimens of *Cloudina hartmannae* from shallow platform carbonates of the upper Gaojiashan member, which may represent the earliest evidence of metazoan predation in the fossil record (Bengtson & Zhao, 1992).

Shallow, high energy facies of the lowermost Dengying Formation at Zhongling show continued euxinia from three samples on the shelf margin (Li et al., 2010), whilst basinal sections of the equivalent Liuchapo Formation (Huanglian and Longbizui) indicate predominance of ferruginous anoxia (Och et al., 2015; Wang et al., 2012). On the shallow platform, decreasing Ce/Ce* has been suggested to indicate gradually more oxygenated conditions (Ling et al., 2013); however, additional RSE and Ce/Ce* data recorded from the subtidal Shibantan member support punctuation of reducing conditions by temporary oxygenation during storm events (Duda et al., 2014). Intermittent increase in local marine sulphate concentration accompanying deposition of the Dengying Formation at the Wuhe‐Yanjiahe section (Hubei) may have been associated with increased salinity and consequent density‐driven stratification during periods of intrashelf basin shallowing and evaporation, similar to conditions suggested during deposition of the underlying Doushantuo member IV at the shallow Xiaofenghe section (Yangtze Gorges, Hubei) (Duda et al., 2014; Hohl et al., 2015).

Earliest Cambrian deposits which locally overly the Dengying Formation on the shallow platform include the Yanjiahe and Zhujiaqing Formations. Yanjiahe Formation deposits occupy present‐day Hubei province, whilst equivalent deposits in Yunnan are assigned to the Zhujiaqing Formation and consist of three minor shallowing cycles of the Daibu, Zhongyicun and Dahai members (Li, Evans, & Halverson, 2013; Och et al., 2015). Continued contemporaneous deposition of basinal Liuchapo Formation cherts and shales is indicated by a U‐Pb age of 536.3 ± 5.5 Ma in the upper Liuchapo Formation at Ganziping (Chen et al., 2009; Och et al., 2015). Iron speciation data of the Yanjiahe Formation and equivalents indicate ferruginous shallow water conditions with at least intermittent euxinia at Jiuqunao, whilst deposition of the upper Liuchapo Formation continued under sustained ferruginous anoxia at basinal Huanglian and Longbizui (Och et al., 2015; Wang et al., 2012).

Approximate equivalence between overlying Cambrian Shiyantou and Shuijingtuo Formations is inferred from zircon U‐Pb ages of 526.5 ± 1.1 Ma (Compston et al., 2008) and 526.4 ± 5.4 Ma (Okada et al., 2014), respectively. A minimum U‐Pb SHRIMP age of 532.3 ± 0.7 Ma has been assigned to the basal Niutitang Formation at Zhongnan (Jiang et al., 2009) and dating of overlying units of the Niutitang Formation give a U‐Pb age of 524.2 ± 5.1 Ma from Panmen section, Guizhou province (Chen, Ling, et al., 2015) and a composite Re‐Os age of 521 ± 5 Ma from three sections of Hunan and neighbouring Guizhou province (Xu, Lehmann, Jingwen, Wenjun, & Andao, 2011). Together, these ages indicate at least partially contemporaneous deposition of the Niutitang Formation with the shallow marine equivalent Shiyantou and Shuijingtuo Formations.

Global sea‐level rise during the early Cambrian (Haq & Schutter, 2008) is expressed in deepening deposits of the Shiyantou, Shuijingtuo and lower Niutitang Formations across sections of the Yangtze Platform and basin, with evidence for dominant ferruginous anoxia interrupted by periods of extensive euxinia (Canfield et al., 2008; Feng, Li, Huang, Chang, & Chu, 2014; Och et al., 2013, 2015; Wang et al., 2012). Equivalent middle and upper Niutitang shales show first evidence from iron speciation for at least episodic oxygenation of the outer shelf and shelf margin at Jinsha and Yangjiaping, and basin at Longbizui which has been attributed to progressive deepening of the oxycline (Feng et al., 2014; Jin et al., 2016; Wang et al., 2012). Cherts of the deep basin equivalent Hetang Formation continue to indicate ferruginous conditions at Diben (Yuan et al., 2014). The earliest definitive evidence for oxia inferred from iron speciation within the shallow platform at Xiaotan is found within the upper Yu'anshan Formation, which is accompanied by continued anoxia of the Minxinsi Formation at Weng'an implying continued water column redox stratification (Jin et al., 2016; Och et al., 2015).

An effective Fe‐Mn oxide shuttle has been proposed as a mechanism for producing observed differences in preserved δ^98^Mo between sections of the early Cambrian South China Block (Cheng et al., 2016; Li, Meng, et al., 2015). Under this model, adsorption of ^95^Mo onto Mn oxides in well‐oxygenated, nearshore surface waters and re‐release during reductive dissolution in the underlying zone of Fe‐Mn reduction was followed by quantitative scavenging within euxinic levels of the water column consistent with Mo cycling in modern euxinic environments (Algeo & Tribovillard, 2009; Cheng et al., 2016; Kendall et al., 2015; Li, Meng, et al., 2015).

Early Cambrian biota of the Yangtze Block include small shelly fossil assemblages preserved in shallow and deep‐water facies of the Zhujiaqing and Kuanchuanpu Formations, succeeded by SSFs of the Shiyantou Formation (Jin et al., 2016). These assemblages give way to early trilobites, articulated sponges and bivalved arthropods of the Niutitang sponge fauna within outer shelf fine‐grained siliciclastic deposits of the Niutitang Formation (Jin et al., 2016). Weakly phosphatised putative animal embryos have also been documented from the shallow shelf mixed ferruginous/euxinic Shuijingtuo Formation at Wengzishi section, Hubei Province (Broce, Schiffbauer, Sen Sharma, Wang, & Xiao, 2014). The increasing proportion of oxic samples recorded from Cambrian Stage 3 appears to be accompanied by increased ecosystem complexity throughout shallow shelf to outer slope environments of the Yangtze Block. However, these diverse assemblages dominantly comprise motile benthic communities, including trilobites for which occasional exploration of dominantly anoxic deeper slope environments (e.g., Songtao) may have been permitted during fleeting oxygenation (Feng et al., 2014; Vannier, García‐Bellido, Hu, & Chen, 2009).

#### 4.1.2 Laurentia

Sediments along the Canadian Cordillera were deposited in a rift setting, with evolution to a passive continental margin and associated subsidence initiated in the mid‐Ediacaran (MacDonald et al., 2013; MacNaughton, Narbonne, & Dalrymple, 2000; Yonkee et al., 2014). Ediacaran age deposits of the Windermere Supergroup outcrop at Goz Creek in the Wernecke Mountains of Yukon, Canada (Figure 4a) (Johnston et al., 2013) and are complemented by an expansive record from the deeper Sekwi Brook section of the Mackenzie Mountains (Figure 4b). Detailed multiproxy geochemical analyses of both sections have enabled geochemical scrutiny of the marine environment during deposition of the fossiliferous June Beds and Blueflower Formations (Johnston et al., 2013; Sperling, Carbone, et al., 2015). Regional carbon isotope chemostratigraphic correlation and reconstruction of relative sea‐level change within the Gametrail, Blueflower and Risky Formations suggest deposition along an unrestricted slope to basin environment (MacDonald et al., 2013; MacNaughton et al., 2000).

##### North Canadian Cordillera

###### Goz Creek, Wernecke Mountains 

Iron speciation at Goz Creek (Figure 4a) reveals almost continuous anoxic ferruginous deposition within the Ediacaran Windermere Supergroup, with a brief oxic interval recorded during regression within the deep‐water upper Sheepbed Formation (Johnston et al., 2013). Oxia of the upper Sheepbed Formation has also been documented at the siliciclastic Shale Lake section (after stratigraphic re‐evaluation by MacDonald et al., 2013). An observed trend towards heavier δ^34^S_py_ documented throughout the Sheepbed Formation has been interpreted to represent pore water sulphate limitation (Johnston et al., 2012).

A first‐order shallowing trend is superimposed upon a number of parasequences within the overlying Blueflower Formation yet no change in dominant redox is recorded, with all iron speciation samples suggesting anoxic ferruginous conditions. Proximal deposition of the shallow water Blueflower Formation is accompanied by the potential for estuarine trapping of iron oxides, which is suggested to account for a shift towards higher ratios of Fe_HR_/Fe_T_, thus yielding a false anoxic signal (Johnston et al., 2013).

###### Sekwi Brook, Mackenzie Mountains 

Stratigraphy of Sekwi Brook records a deeper water environment than that of Goz Creek, with similarly unrestricted access to the open ocean (Sperling, Carbone, et al., 2015). Iron speciation and trace element analyses of the June Beds and Blueflower Formation (Figure 4b) are in broad agreement with the results of Johnston et al. (2013) at Goz Creek, where no clear progression towards more persistent oxygenation across the sampled interval is detected (Sperling, Carbone, et al., 2015). Outcrops at the base of the section have been tentatively correlated to the deep‐water Sheepbed Formation deposited during one cycle of relative sea‐level change and show evidence for intermittently oxic conditions within both the transgressive and highstand systems tracts. Overlying fossiliferous shales of the June Beds show overwhelming evidence for protracted anoxic ferruginous conditions, punctuated by two brief episodes of oxia initially within the middle lowstand systems tract, and again during transgression nearing the top of the June Beds. The exclusively carbonate sequence of the Gametrail Formation was not sampled at Sekwi Brook; however, iron speciation of the overlying Blueflower Formation again indicates predominance of anoxic ferruginous conditions with two minor oxic intervals, both of which immediately follow maximum flooding surfaces (Sperling, Carbone, et al., 2015).

Soft‐bodied biota, which inhabited the Laurentian passive margin outcrop in the June Beds and Blueflower Formations of the Windermere Supergroup at Sekwi Brook, represent an Avalon‐type fossil assemblage with abundant rangeomorphs and arboreomorphs, in addition to the erniettamorph *Namalia* (Narbonne et al., 2014; Sperling, Carbone, et al., 2015). The Avalon assemblage of the Windermere Supergroup is primarily preserved on the soles of mass‐flow deposits; however, some specimens are preserved in three dimensions similar to those of the Nama Group, Namibia (Narbonne et al., 2014). This facies control on fossil occurrence complicates identification of first appearance within the June Beds (Sperling, Carbone, et al., 2015).

Whilst the June Beds biotic assemblage inhabited a deep‐water lower slope setting, facies of the upper Blueflower Formation represent a lower shoreface to offshore environment and incorporate specimens of the new tubular genera *Sekwitubulus annulatus* and the larger flexible *Annulatubus flexuosus*, in addition to a shallow water dickinsonid *Windermeria aitkeni* (Narbonne et al., 2014).

The majority of samples from the Sekwi Brook section have depleted Fe_T_/Al below the lower calibrated threshold value expected under normal marine deposition (Raiswell et al., 2008), similar to the basal Sheepbed and Blueflower Formations of Goz Creek (Johnston et al., 2013). Whilst previous studies have advised caution in interpretation of shales exhibiting extreme depletions (e.g., Sahoo et al., 2012), one hypothesis provided by Sperling, Carbone, et al. (2015) concerns the threshold value itself and suggests the potential for significantly different balances of iron and aluminium cycling in the Ediacaran which may be supported by the closer to “normal” shale Fe/Ti ratios.

##### South Canadian Cordillera

###### Cariboo Mountains, British Columbia 

Ediacaran deposits of southern Canadian Cordillera outcrop in the Cariboo Mountains of British Columbia and represent post‐rift basinal deposition within the Laurentian passive margin (Canfield et al., 2008; Ross, Bloch, & Krouse, 1995; Schwarz & Arnott, 2007). A maximum age of 608 ± 4.7 Ma is given from Re‐Os dating of black shales of the Old Fort Point Formation (Kendall, Creaser, Ross, & Selby, 2004) and a Re‐Os age of 632.3 ± 6.3 Ma has been documented from the lower Sheepbed Formation (Rooney, Strauss, Brandon, & Macdonald, 2015). Deposition of the upper Kaza and Isaac Formations is tentatively considered to have taken place contemporaneous to deposition of Sheepbed and overlying formations (MacDonald et al., 2013; Rooney et al., 2015; Yonkee et al., 2014). Despite difficulties in correlation, iron speciation data recorded in Canfield et al. (2008) and Dahl et al. (2010) allow for a snapshot of approximately time‐equivalent lower slope to basinal water column conditions (Figure 4c) (Ross et al., 1995). Mudstones and shales of the Upper Kaza Formation were deposited in a quiescent basinal environment and indicate exclusively oxic conditions (Canfield et al., 2008; Dahl et al., 2010). In contrast, the overlying Isaac Formation contains abundant rapidly deposited turbidites, debris flows and slumping consistent with lower slope deposition (Schwarz & Arnott, 2007). Ratios of Fe_HR_/Fe_T_ which fall below the calibrated upper threshold for oxic conditions may be an expected consequence of rapidly deposited sediments; however, the mean value of Fe_HR_/Fe_T_ of Isaac Formation samples, which fall below this threshold, is ~0.11, indicating significant Fe_HR_ depletion. At least intermittent oxia is implied with a possible trend towards ferruginous anoxia up‐section.

###### Additional sections of the Laurentian passive margin 

The Upper Miette Group of the Windermere Supergroup in southeastern British Columbia and Wood Canyon of Death Valley also host Ediacaran fossils of both soft‐bodied and biomineralising organisms. The Upper Miette Group is tentatively considered chronostratigraphically equivalent to shallow marine carbonates of the Cunningham Formation which locally overlies the Isaac Formation of the Southern Canadian Cordillera (Ross et al., 1995). The Byng carbonate platform within the Upper Miette Group contains *Cloudina* and *Namacalathus,* and shallow water siltstones of the potentially coeval Yellowhead carbonate platform preserve soft‐bodied forms which include *Cyclomedusa* found in association with stromatolitic mounds (Hofmann & Mountjoy, 2001).

The upper Stirling and lower Wood Canyon Formations of California preserve an assemblage strikingly similar to that of the upper Nama Group within a subtidal, possible‐deltaic shallow marine succession with *Cloudina* alongside *Ernietta* and *Swartpuntia* (Corsetti & Hagadorn, 2000). Additionally, the earliest recorded example of the ichnofossil *Zoophycos* is recorded from the overlying lower Cambrian portion of the Wood Canyon Formation (Sappenfield, Droser, Kennedy, & McKenzie, 2012). These additional sections have not yet been evaluated using redox proxy methods and so present an intriguing succession for future geochemical consideration.

#### 4.1.3 Nama Group

Exceptional exposure along two shelf‐to‐basin transects has allowed for sequence stratigraphic reconstruction, geochemical analysis and fossil distribution of a substantial portion of the Nama Group down to parasequence level (Figure 5) (Dibenedetto & Grotzinger, 2005; Saylor, 2003; Saylor, Grotzinger, & Germs, 1995; Saylor, Kaufman, Grotzinger, & Urban, 1998; Wood et al., 2015). The Nama foreland basin formed on the Kalahari Craton as a consequence of convergence along the Damara and Gariep orogenic belts to the present northeast and southwest, respectively, due to closure of the Brazilides Ocean during amalgamation of southwest Gondwana (Gaucher, Frimmel, & Germs, 2009). The Nama basin was subdivided into northern Zaris and southern Witputs sub‐basins by a zone of depositional thinning across the “Osis Arch” palaeobathymetric high (Germs, 1983). Correlative formations of fluvial to shallow marine siliciclastic and carbonate sediments within both sub‐basins have been mapped extensively across the Osis Arch and support basin connectivity during deposition, with a general palaeodepth increase to the northwest in the Zaris sub‐basin and southwest in the Witputs sub‐basin (Germs, 1983). Deposits of both sub‐basins are subdivided into the lower Kuibis Subgroup, and the upper Schwarzrand Subgroup, with diachronous deposition of lower Nama Group sediments recording a marine transgression onto the underlying Proterozoic basement. Whilst thinning across the Osis Arch during deposition of the Kuibis Subgroup may suggest the possibility for minor independence of local water column conditions between the two sub‐basins, the extent of thinning gradually decreases with deposition of the overlying Schwarzrand Subgroup indicating a trend towards more pronounced connectivity corresponding to an overall marine transgression nearing the Ediacaran‐Cambrian boundary (Germs, 1983; Saylor et al., 1995). Unrestricted connection of the Nama basins with the Brazilides Ocean to the present‐day west is implied by near‐primary record of δ^13^C_carb_. This record presents major features in agreement with all other time‐equivalent sections globally, showing an increasing positive trend from values as low as −7.40‰ within deep‐water deposits of the Kanies member of the Dabis Formation (Kuibis Subgroup), and becoming positive during deposition of the lower Omkyk member of the Zaris Formation (Kuibis Subgroup) potentially relating to transitional recovery from the global Shuram‐Wonoka negative δ^13^C_carb_ anomaly (Kaufman, Hayes, Knoll, & Germs, 1991; Wood et al., 2015).

Uranium‐lead chronology of four volcanic ash beds has constrained the duration of Nama Group deposition, with a lower Hoogland member (upper Kuibis Subgroup) age of 547.32 ± 0.65 Ma, and an age of 540.61 ± 0.67 Ma for the upper Spitskop member of the Urusis Formation (Schwarzrand Subgroup), coincident with the first appearance datum of the trace fossils *T. pedum* and *S. narbonnei* (Grotzinger, Bowring, Saylor, & Kaufman, 1995; Jensen & Runnegar, 2005; Schmitz, 2012). Strata of the Dabis Formation and lower Zaris Formation which were deposited prior to the lowermost ash bed rest atop crystalline basement along a dramatic, readily identifiable angular unconformity with an contact age inferred at ~550–553 Ma, whilst conglomeratic and fluvial to shallow marine siliciclastic facies of the Nomtsas Formation unconformably overlie the Urusis Formation with an intercalated ash bed dated at 538.18 ± 1.11 Ma (Grotzinger et al., 1995; Schmitz, 2012). Therefore, total stratigraphic coverage of the Nama Group below the Cambrian Fish River Formation spans the final 10–12 million years of the Ediacaran Period (Figure 5).

First appearance of soft‐bodied Ediacara biota belonging to the Nama assemblage is documented from proximal sandstones of the Kanies Member, Dabis Formation (Bouougri, Porada, Weber, & Reitner, 2011). Subsequent likely in situ occurrence of *Ernietta, Rangea* and *Nemiana* is documented within layers of the upper Kliphoek (Aar) Member at Arasab and Grens Farm sections (Hall et al., 2013; Wood et al., 2015). Nama assemblage fossils are recorded throughout the overlying Schwarzrand Subgroup of the Witputs sub‐basin and include the Erniettamorpha *Pteridinium, Rangea* and *Paramedusium*, in addition to discoidal *Cyclomedusa* in the lower Schwarzrand Subgroup (Germs, 1995). *Pteridinium*,* Swartpuntia*,* Aspidella* and *Bradgatia* are also recorded within the Spitskop Member at Swartpunt section (Grotzinger et al., 1995; Narbonne et al., 1997). The published range of soft‐bodied fossils within contemporaneous deposits of the Zaris sub‐basin is restricted to the Nudaus Formation of the lower Schwarzrand Subgroup and includes recently documented occurrences of *Aspidella* and *Shaanxilithes* (Darroch et al., 2016), in addition to mention of possible *Pteridinium* (Grotzinger et al., 1995).

The Omkyk and Hoogland Members of the Zaris Subgroup and the Huns and Feldschuhhorn Members of the Urusis Formation host prominent thrombolite‐stromatolite reefs, including the thick transgressive pinnacle reef succession at Driedoornvlagte, mid‐ramp bioherms and biostromes of the highstand systems tract at Zebra River and pinnacle reefs which formed during transgression of the upper Huns and middle Felschuhorn Members (Grotzinger, 2000; Saylor et al., 1995). The earliest documented occurrences of *C. hartmannae, Cloudina riemkeae* and *N. hermanastes* in the Nama Group are found in association with thrombolitic‐stromatolitic microbial reefs of the lower Omkyk Member, and the first appearance of active reef‐building by a metazoan is found in the high energy mid‐ramp setting at Driedoornvlagte (Grotzinger, 2000; Penny et al., 2014; Wood & Curtis, 2015). Driedoornvlagte also exhibits the only known occurrence of the neptunian dyke‐dwelling, robust skeletal *Namapoikia rietoogensis* of probable poriferan affinity (Wood et al., 2002). Associated *Cloudina* and *Namacalathus* have also been found within shallower facies of the upper Omkyk and lower Hoogland members at Zwartmodder (Wood et al., 2015) and ichnofossil‐rich strata immediately overlying a soft‐bodied Nama assemblage horizon in the middle Spitskop Member at Swartpunt (Darroch et al., 2015; Narbonne et al., 1997; Wood et al., 2015).

A wealth of trace fossil evidence recorded throughout the Nama Group initially enabled its interpretation as a terminal Ediacaran/Vendian succession (Crimes & Germs, 1982). Of particular note are vertical biogenic trace fossils within the lower Nudaus Formation of the Zaris sub‐basin and basal Huns member of Urusis Formation in the Witputs, *Treptichnus*‐like trace fossil of the basal Huns members and *S. narbonnei* from Spitskop Member (Jensen & Runnegar, 2005; Jensen, Saylor, Gehling, & Germs, 2000; Wilson et al., 2012). In addition, an array of enigmatic tubular compression fossils have been noted from the lowermost Nudaus Formation and Feldschuhhorn member of the Witputs sub‐basin (Cohen et al., 2009), and a diverse assemblage of organic walled microfossils has been noted from the Schwarzrand Subgroup of the Witputs sub‐basin including laeosphaerid acritarchs and *Vendotaenia* (Germs, Knoll, & Vidal, 1986).

Recent, extensive redox analyses utilising iron speciation of shales, silts and carbonates and Fe_T_/Al ratios of nine study sections within the Nama Group in association with palaeoecological data, has enabled reconstruction of three distinct time‐equivalent shelf‐to‐basin transects, thus allowing for interpretation of the relationship between redox hospitability and sustained ecological presence (Figure 5) (Sperling, Wolock, et al., 2015; Wood et al., 2015). The first transect incorporates data of the Dabis Formation from three sections of the Witputs sub‐basin at Arasab, Grens and Zuurburg, and two sections of the Zaris sub‐basin at Zwartmodder and Brak. Shallow water oxia persisted throughout deposition of the Kanies member (Zwartmodder) and lower Mara member (Zuurburg) coincident with considerable redox heterogeneity between the moderately deeper Arasab and Grens sections which record probable oxic conditions (inferred from extremely low Fe_T_ of carbonate sediments) and ferruginous anoxia (Wood et al., 2015). Deep‐water deposition within the Zaris sub‐basin (Brak) is seen to have been exclusively anoxic and ferruginous (Wood et al., 2015). Three shallowing‐upward cycles are associated with deposition of the Dabis Formation within the Witputs sub‐basin, whilst only one transgressive unit comprising the Kanies member is recognised within the Dabis Formation of the Zaris sub‐basin.

The second transect includes sections comprising time‐equivalent carbonate platform deposits of the Omkyk, Hoogland and Urikos members of the Zaris Formation (Zaris sub‐basin) on farms Zwartmodder, Omkyk, Zebra River and Driedoornvlagte. Throughout the Omkyk and Hoogland Members, a highly heterogeneous redox environment is evident with shallow waters at Omkyk characterised by repeated establishment of ferruginous anoxia punctuating dominant probable oxic conditions (Wood et al., 2015). Extensive growth of the mid‐ramp microbial‐metazoan reef at Driedoornvlagte occupied a transgressive systems tract of the upper Omkyk Member (Unit 3 m, OS2, Figure 5) (Adams, Schroder, Grotzinger, & McCormick, 2004). Importantly, new data suggest that reef growth at Driedoornvlagte which took place during long‐lived, probable oxic transgression, was terminated with development of reducing conditions which accompanied blanketing by shales of the deep‐water Urikos Member. Similarly oxic conditions which appear to have dominated time‐equivalent deposition during the second major cycle of the Omkyk Member at Zebra River are accompanied by growth of microbial‐metazoan biostromes which dominantly grew during highstands (Adams et al., 2005). Pulsed ferruginous conditions are recorded during OS2 at Zebra River, which may either reflect development of sluggish circulation within a more proximal and productive inner‐ramp position, or may imply upwelling of anoxic deep water during transgression (Wood et al., 2015). Indeed, ferruginous conditions which accompanied deposition of transgressive Urikos Member shales during Unit 3 m on the deep, outer shelf at Driedoornvlagte are also seen to have developed at Zebra River. However, whilst the carbonate factory at Driedoornvlagte was terminated through inundation by high clastic flux of the Urikos Member, contemporaneous ventilated and likely oligotrophic conditions at Zebra River were accompanied by sustained oxia and successful repetitive development of thrombolitic‐stromatolitic biostromes, accompanied by both *Namacalathus* and *Cloudina*. Equivalent deposits of the lowermost Zaris Formation in the Witputs sub‐basin have been sampled at Arasab and also indicate probable oxic water column conditions within the Mooifontein Member (Wood et al., 2015).

The third transect incorporates three sections of the Feldschuhhorn and Spitskop Members of the upper Urusis Formation at the mid‐ramp pinnacle reefs locality, outer ramp FSH section and variable depth deposits at Swartpunt (Sperling, Wolock, et al., 2015; Wilson et al., 2012; Wood et al., 2015). Additional redox data of Nomtsas Formation deposits at the distal Sonntagsbrunn section are also considered herein (Sperling, Wolock, et al., 2015; Wilson et al., 2012). All sections of transect 3 show a dominantly oxic marine environment within the Witputs sub‐basin across the Precambrian‐Cambrian boundary; however, two brief intervals of ferruginous anoxia recorded from the Spitskop member at Swartpunt (Wood et al., 2015) may be suggestive of continued water column redox stratification. Accommodation increase during transgression appears to have favoured growth of pinnacle reefs capping the Huns Member on Swartkloofberg Farm similar to conditions at Driedoornvlagte, although there is little indication of anoxic stress present within the Witputs sub‐basin at this time, and cessation of reef growth is seen to coincide with repeated drowning by siliciclastic deposits of the Feldschuhhorn Member (Grotzinger, 2000; Saylor et al., 1995).

Recent complimentary data supporting redox stratification of the Nama Basin have enabled nuanced interpretation of intermediate redox states through identification of unusual REE(+Y) profiles (Tostevin, Wood, et al., 2016). This has also allowed infilling of data gaps where Fe_T_ < 0.5 wt% of some carbonate samples previously impeded analysis via Fe speciation. Where iron speciation indicates surface water oxia, these data are corroborated by negative Ce anomalies. However, where highly reactive iron enrichments indicate anoxic ferruginous conditions, REE patterns either show the absence or expression of positive Ce anomalies (Tostevin, Wood, et al., 2016). In the latter case, positive Ce anomalies are interpreted to indicate the presence of an intermediate layer of dissolved oxygen ≥~10 μm immediately overlying ferruginous deeper waters (Tostevin, Wood, et al., 2016). Within this layer, the reductive dissolution of Mn (oxyhydr)oxides likely resulted in release to the water column of Ce(IV), leading to Ce accumulation and resultant enrichment in carbonate sediments relative to neighbouring REEs (Tostevin, Wood, et al., 2016). In the Nama Group, Ce anomaly data indicate episodic incursion of the manganous zone at shallow water Arasab, Grens, Zwartmodder and Omkyk sections and intermediate depth at Zebra River. However, an absence of positive Ce anomalies at Driedoornvlagte, the Pinnacle Reefs or Swartpunt sections supports sediment deposition at these localities under predominantly oxic conditions (Figure 7a) (Tostevin, Wood, et al., 2016).

Whilst almost exclusive oxia recorded within the upper Urusis Formation of the Witputs sub‐basin may suggest progressive oxygenation of the Nama Group towards the Ediacaran‐Cambrian boundary (Wood et al., 2015), additional sampling of Urusis Formation strata of the Zaris sub‐basin appears to complicate this development. Fine‐grained, olive green and purple‐red mudstones are interbedded with channelised sandstones of the Schwarzrand Subgroup in the Zaris sub‐basin and yield iron speciation data which indicate exclusive formation under anoxic ferruginous water column conditions. Ratios of Fe_T_/Al suggest normal marine deposition within the calibrated range of 0.53 ± 0.11, with the exception of 5 outliers indicating significant iron enrichment and supporting deposition under an anoxic water column (Figure 5).

##### Redox evolution accompanying the earliest reef‐builders

The majority of sampled sections within the Kuibis Subgroup represent shallow to mid‐ramp marine facies above storm wave base (with the exception of the most distal section at Brak), and as such introduction of oxygen via diffusion and surficial mixing from the overlying atmosphere is expected to have been pervasive. Riverine input of oxic freshwater may also have been an important mechanism for introduction of dissolved oxygen to the nearshore environment, with palaeocurrent data supporting dominantly westward‐directed effluent sediment transport from the Kalahari Craton, evident from trough cross‐stratification within siliciclastic lowstand systems tracts of the Kanies and lower Kliphoek members of the Dabis Formation (Germs, 1983; Saylor et al., 1995).

During deposition of the Dabis Formation, two cycles of marine transgression show repetitive shoaling of the oxycline (Figure 5), with switching between ferruginous and probable oxic signatures at Arasab and Grens, likely representative of retrogradational stacking during higher order parasequences and deposition above the oxycline. Resultant short‐lived exposure to oxic conditions, if required, may have allowed for brief establishment of soft‐bodied biota found throughout this formation in the Witputs sub‐basin. Distal dolomites, limestones and shales at Brak record uninterrupted deposition below the oxycline and represent anoxic ferruginous conditions which dominated the deep marine environment at this time.

Throughout the overlying Zaris Formation, frequent occurrence of anoxia in proximal, shallow environments has been interpreted as a consequence of upwelling anoxic ferruginous deep water, which is supported by progressively decreasing Fe_T_/Al within shallower sections (Wood et al., 2015). Although shallow waters of the exposed mid‐ramp are thought to have been subject to active physical mixing and effective oxygenation, frequent incursions of anoxia are also thought to correspond to upwelling. However, relatively quiescent conditions at proximal Zwartmodder and Omkyk sections conducive to microbial mat growth may represent an environment prone to high surface water productivity fuelled by riverine nutrient input. Efficient remineralisation of the resultant elevated organic matter via aerobic respiration within shallow waters may have reduced dissolved oxygen concentration, followed by reduction of readily available shallow water iron oxides leading to thickening of a shallow water zone of Fe‐Mn reduction (Figure 7a) (Tostevin, Wood, et al., 2016).

Metre‐scale reefs constructed through mutual attachment of *Cloudina* have so far been noted solely from the thrombolite‐stromatolite reef at Driedoornvlagte and grew exclusively during transgression of the Upper Omkyk member (Penny et al., 2014; Wood & Curtis, 2015). Laterally extensive thrombolite‐stromatolite biostromes of the upper Omkyk and lower Hoogland members at the more proximal Zebra River locality are associated with solitary mat‐sticking *Cloudina* and *Namacalathus*. These biostrome horizons record pulsed incursion of anoxic ferruginous deeper waters into a dominantly oxic/probable oxic water column (Wood et al., 2015). It has been shown that unconsolidated seafloor conditions relating to increased siliciclastic influx from the Damara orogen to the north may have prevented early cementation conducive to formation of relief‐structures during platform development of the Hoogland member (Dibenedetto & Grotzinger, 2005) and cessation of *Cloudina* reef growth at Driedoornvlagte is seen to locally occur simultaneously with a transition to ferruginous conditions which accompanied siliciclastics of the Urikos member (Figures 5 and 7b). This may imply a favourable combination of sustained oxic conditions and low sediment influx of the mid‐ramp at Driedoornvlagte, which supported greater diversity of calcifying ecologies. Similarly, Wood et al. (2015) suggest that successful colonisation of shallow marine environments by diverse calcifying palaeocommunities was likely facilitated during periods of relatively stable oxygenation, and also show larger body size association of both *Namacalathus* and *Cloudina* exclusively within prolonged, stable oxic water column conditions throughout the Nama Group.

Iron speciation results of the Schwarzrand Subgroup north of Osis appear entirely incongruent with fully oxic, unrestricted water column conditions within the connected Witputs sub‐basin to the south, where the overall paucity of anoxic conditions may suggest deepening of the oxycline. The Nudaus Formation of the Schwarzrand Subgroup within the Zaris sub‐basin represents a sequence of prodeltaic (Niederhagen Member) to low‐energy shoreline (Vingerbreek Member) mudstones and laminated and channelised sandstones, with deposition in increasingly shallower water towards the east, nearing the top of the sampled section (Germs, 1983; Grotzinger et al., 1995). Analysis of the Niederhagen Member, which locally overlies Hoogland and Urikos sediments, indicates provenance from a relict volcanic island arc within the Damara Belt, which divided the Congo and Kalahari Cratons to the present‐day north/northwest of the Nama Group (Blanco et al., 2011; Germs, 1983). The axis of the carbonate platform within the overlying Urusis Formation was shifted to the deeper, shallow marine environment to the southwest of Osis, and equivalent facies of the smaller Zaris sub‐basin represent proximal siliciclastic deposition (Germs, 1983). The depocenter of the Zaris sub‐basin gradually shifted southwards associated with diminishing influence of the Osis Arch throughout deposition of the upper Schwarzrand Subgroup (Germs, 1983). A potential scenario for some elevated Fe_HR_/Fe_T_ within the Nudaus and Urusis Formations in the Zaris sub‐basin may follow a simple Fe‐trap mechanism, whereby Fe_HR_ is effectively retained within this proximal deltaic environment (Figure 7b) (Johnston et al., 2013). Such a setting would subsequently act as a source for Fe_HR_ via reductive remobilisation to the subjacent water column via oxic or anoxic iron shuttle processes (Lyons & Severmann, 2006; Severmann et al., 2008). This may be supported by a significantly greater contribution of oxide‐bound iron within the highly reactive iron pool of the Schwarzrand subgroup north of Osis, when compared to contemporaneous siliciclastic deposits of the Witputs sub‐basin, and accompanied by “normal shale” values of Fe_T_/Al. Future detailed sampling and associated sedimentological and relative palaeodepth assessment within the Zaris sub‐basin is required to support either an anoxic or false anoxic water column interpretation.

Whilst diverse soft‐bodied and skeletonising biota thrived within the upper Urusis Formation, as well as motile metazoans evident from the diverse ichnofossil record at the oxic Swartpunt locality, the Schwarzrand Subgroup in the Zaris sub‐basin lacks evidence for a comparatively significant biotic presence but for *Pteridinium* (Grotzinger et al., 1995) and recently recorded *Aspidella* and *Shaanxilithes* within the Nudaus Formation (Darroch et al., 2016). This may simply be a consequence of comparatively poor time‐equivalent stratal exposure north of Osis farm, which has, until recently, impeded extensive body fossil recognition. If the interpretation of an episodic prodeltaic iron‐trap within this formation is correct, then original water column conditions may in fact have been at least intermittently oxic.

#### 4.1.4 Avalonia

Siliciclastic units of the Conception and St. Johns Groups of Newfoundland record deposition in an unrestricted deep‐water environment with rapid emplacement of volcanic ash preserving a wealth of body fossils. The Avalon assemblage of Newfoundland includes rangeomorphs (*Rangea, Charnia,* and *Fractofusus*), possible triradialomorphs (e.g., *Triforillonia costellae*), arboreomorphs (*Charniodiscus*), discoidal fossils (*Aspidella* and *Cyclomedusa*) and possible sponges (*Thectardis*) (Laflamme et al., 2013; Liu et al., 2015; Sperling et al., 2011). Ediacaran organisms which thrived in the basinal waters of Avalonia did so well below the photic zone, similar to conditions which persisted during deposition of the June Beds and Blueflower Formation of the Windermere Supergroup (Narbonne et al., 2014).

A strikingly sharp, early transition from ferruginous anoxia of the deep‐water Mall Bay and Gaskiers axial turbidite sequence to almost continuously uninterrupted oxia of overlying strata is revealed by iron speciation at 11 localities on the Avalon peninsula, which combine to form a 6 km snapshot equating to ~15 Myr of Ediacaran deposition transcending the Gaskiers glaciation (Figure 6a) (Canfield, Poulton, & Narbonne, 2007). The basal Drook Formation defines the boundary for this transition, followed by the first fossil evidence of soft‐bodied Ediacara biota in the fossil record within the upper Drook Formation at approximately 570.94 ± 0.38 Ma (Pu et al., 2016). Persistence of oxia accompanied continued biological establishment in these deep basinal sediments throughout deposition of the overlying Briscal, Mistaken Point, Trepassey and Fermeuse Formations, with just two minor periods of ferruginous anoxia recorded from the base and top of deep delta front deposits of the Fermeuse Formation (Canfield et al., 2007). Whilst the rapid emplacement of turbiditic sediments may innately result in reduced Fe_HR_ accumulation beneath an anoxic water column, the upper layers of each turbidite were sampled and define the finest sediment emplaced at the slowest rate (Canfield et al., 2007). Shale of the Conception and St. Johns Groups are confidently regarded to record oxic deposition within and above the Drook Formation (Canfield et al., 2007).

Lower rates of BSR indicated by low C and S concentrations and persistently elevated δ^34^S_py_ nearing the approximated contemporaneous composition of seawater sulphate are seen to have preceded the Gaskiers Formation (Canfield et al., 2007; Fike et al., 2006). A decrease to predominantly negative δ^34^S_py_ values is subsequently observed in the Drook Formation, coincident with inferred oxygenation of the deep marine environment, followed by a return to more elevated δ^34^S_py_ during deposition of the Fermeuse Formation.

#### 4.1.5 East European Platform

Whilst no direct fossil identification beyond microfossil evidence is possible from drill core samples, the stratigraphic succession from which the Kel'tminskaya‐1 core was extracted is known to host a richly fossiliferous example of the White Sea assemblage, which may have existed approximately contemporaneous with the mid‐upper Doushantuo Formation, Drook Formation and June Beds (Boag et al., 2016). Large ornamented microfossil assemblages composed of acanthamorphic acritarchs have made useful biostratigraphic indicators within the Vychegda Formation (Vorob'eva, Sergeev, & Knoll, 2009), whilst Ediacara biota including the probable motile early molluscan organism *Kimberella* are documented from the overlying Redkino Formation (Fedonkin et al., 2007; Gehling et al., 2014; Martin et al., 2000). The equivalent Ust’‐Pinega Formation on the Onega River also hosts soft‐bodied *Swartpuntia, Vendoconularia triradiata* and *Ventogyrus* (Ivantsov & Fedonkin, 2002; Fedonkin and Ivantsov, 2007).

Redox evolution of the unrestricted EEP (Figure 6b) margin has been evaluated through application of Fe speciation, alongside δ^34^S_py_, δ^13^C and major element analyses of the Kel'tminskaya 1 drillcore (Johnston et al., 2012). The lower 2,000 m of the drillcore comprises mixed siliciclastic and shallow marine carbonate platform deposits of the Cryogenian Yskemess and Vapol’ Formations, which indicate deposition under dominantly anoxic ferruginous conditions with some evidence for infrequent fleeting oxia. Ediacaran siliciclastics of the Vychegda, Redkino and Kotlin Formations unconformably overlie the Vapol’ Formation, with age constraint dictated by microfossil biostratigraphy of basal Vychegda (Vorob'eva et al., 2009) and U‐Pb dating within the upper and lower Redkino Formation (Grazhdankin, 2003; Martin et al., 2000). The lower boundary of the fossiliferous Redkino Formation has since been re‐interpreted by Grazhdankin, Marusin, Meert, Krupenin, and Maslov (2011) to be in the range 570–600 Ma, thereby placing it in approximate stratigraphic equivalence with the Drook Formation of Newfoundland. The underlying Vychegda Formation occupied a mid‐shelf depositional environment and iron speciation measurements suggest predominantly oxygenated conditions during deposition with minor incursions of ferruginous anoxia (Johnston et al., 2012). Additional δ^34^S_py_ data from the Ediacaran Vychegda Formation reveal generally depleted values, supporting the existence of an oxidative water column sulphur cycle (Johnston et al., 2012). The overlying 1,000 m siliciclastic succession of the Redkino and Kotlin Formations exhibits exclusive oxia, recorded by low Fe_HR_/Fe_T_. Higher δ^34^S_py_ observed in deposits of the Redkino and Kotlin successions is considered to be a consequence of localised sedimentary pore water sulphate limitation (Johnston et al., 2012). These data have been interpreted to represent a shift towards oxygen stability of the local environment reflected in reduced variation of Fe_HR_/Fe_T_ about the mean up‐section (Johnston et al., 2012). This is comparable to deep‐water sediments of Avalonia which may indicate that oxygen concentration allowed for effective suppression of anoxia in the local water column from as early as 570–600 Ma (Canfield et al., 2007).

#### 4.1.6 Arroyo del Soldado Group

Outcropping Ediacaran stratigraphy of the Río de la Plata craton in Uruguay constitute the Arroyo del Soldado Group, which was unconformably deposited over Archean and Proterozoic units of the Nico Perez Terrane and includes the Barriga Negra, Yerbal, Polanco, Cerro Espuelitas, Cerros San Francisco and Cerro Victoria Formations (Blanco, Rajesh, Gaucher, Germs, & Chemale, 2009; Gaucher, 2000; Gaucher, Frimmel, & Germs, 2009). Whilst dating of the Arroyo del Soldado Group has been complicated by the sparsity of zircon‐bearing ash beds, recent stratigraphic reappraisal has positioned deposits of the Barriga Negra Formation below or equivalent to the lower Yerbal Formation (Gaucher, Frei, Sial, & Cabrera, 2011), constraining a maximum detrital zircon age for deposits of the Lower Yerbal Formation of 566 ± 8 Ma (Blanco et al., 2009; Gaucher et al., 2008).

Fossils of the Arroyo del Soldado Group include two distinct acritarch assemblages, in addition to the biomineralising probable animal *Cloudina* (Gaucher, 2000). Examples of in situ haematised *C. riemkeae* predominantly outcrop in upper Yerbal Formation siltstones and reworked fragments of *C. riemkeae* have been reported from within storm deposits of the overlying Polanco Formation (Gaucher, 2000; Gaucher & Poiré, 2009).

Iron speciation analyses of the Yerbal and Polanco Formations (Figure 6c) indicate the predominance of anoxic ferruginous water column conditions with some evidence for occasional deposition in oxic waters (Frei, Gaucher, Stolper, & Canfield, 2013). Additional RSE and Ce/Ce* anomaly data of the Arroyo del Soldado Group are consistent with iron speciation data and are interpreted to indicate a water column with suboxic to anoxic non‐sulphidic depths, overlain by an oxygenated surface layer (Aubet et al., 2012; Pecoits, 2010).

## 5 Discussion

### 5.1 Palaeogeographic controls on local redox

**Figure 8:**
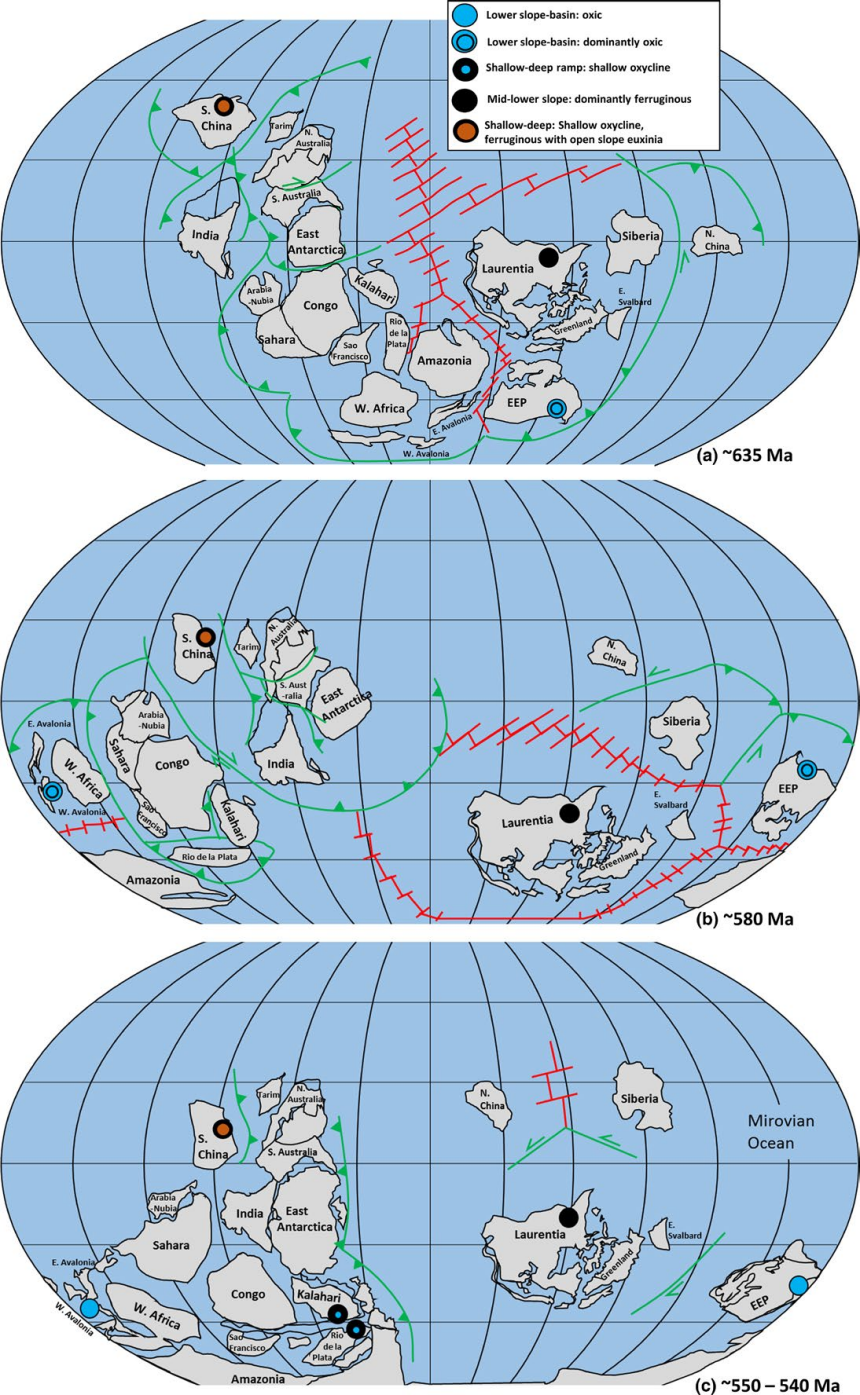
(a–c) Schematic illustration of evolving local redox environments throughout the Ediacaran (635–540 Ma). Modified after Li et al. (2013). (a) 635 Ma. Sections of varying depth across the Yangtze platform and basin (South China) indicate predominance of anoxia with euxinia restricted to mid‐lower slope. Lower slope of the Laurentian passive margin (Northwest Canada) shows ferruginous anoxia and the lower Vychegda Formation (East European Platform) shows predominance of oxia with occasional ferruginous anoxia. (b) 580 Ma. Avalonia and the East European Platform occupy similar mid‐latitude position. Iron speciation data from Western Avalonia (Newfoundland) shows predominantly oxic conditions on the lower slope. Short‐lived oxia recorded from sections of Laurentia. South China Block continues to migrate to low latitude (Zhang et al., 2015). (c) 540 Ma. Shallow to mid‐depth sections of the Kalahari (Namibia) and Río de la Plata Cratons (Uruguay) indicate continued intermittent ferruginous anoxic incursions [Colour figure can be viewed at wileyonlinelibrary.com]

The beginning of the Ediacaran saw the waning stages of breakup of the supercontinent Rodinia and climatic recovery following the global Marinoan glaciation, and the ensuing 95 million years witnessed substantial migration of isolated cratons across a wide variety of latitudinal ranges during assembly of Gondwana (Figure 8). Consideration of dominant redox conditions recorded within each environment, alongside the extent of local restriction, allows speculation as to the possible control of latitudinal position on palaeoredox evolution throughout this interval.

Gradual equatorial migration of the South China Block (Li et al., 2013; Zhang et al., 2015) is associated with little change in dominant redox condition, with anoxia documented into the early Cambrian (Figure 3). In fact, sections of the Yangtze Block are exceptional in that they include the only documented development of spatially extensive and sustained euxinia within Ediacaran environments (Figure 7a–c). This implies elevated seawater sulphide relative to reactive iron which has been variably attributed to local hydrothermal activity, riverine delivery and elevated productivity‐induced anoxia within a sulphate replete open shelf environment (Chen et al., 2009; Och et al., 2015; Sahoo et al., 2016; Wang et al., 2012). The evolution of redox within intrashelf basins of the Yangtze Block was likely associated with the combined effects of variable sulphate supply, nutrient delivery and local water column stagnation. However, maintenance of euxinia on the freely connected slope requires persistently elevated productivity. Primary fluid inclusion analyses of halite from the Dengying Formation indicate an approximate paleo‐seawater temperature close to that of the modern tropics during the final 10 million years of Ediacaran deposition (Meng et al., 2011). With lowered solubility of dissolved O_2_ under higher water temperatures, the approximately equatorial paleo‐latitude of the South China Block at 550–540 Ma may in itself have been less susceptible to extensive water column oxygenation under the lower atmospheric oxygen concentrations of the Ediacaran Period. Although there is some evidence for increased oxygenation across the Yangtze Block, oxic conditions recorded in Ediacaran sections (e.g., Baiguoyuan) are largely confined to shallow platform sediments intermittently deposited within the oxygenated surface mixed layer.

Deep slope deposits of Laurentia also record persistent anoxia within the lower Sheepbed Formation at ~635 Ma, with only intermittent oxic influence documented through to the terminal Ediacaran upper Risky Formation (Johnston et al., 2013; Sperling, Carbone, et al., 2015). A low latitude position favourable to Ekman‐induced surface water transport away from the Laurentian continental margin may have resulted in persistent upwelling which sustained deep‐water anoxia through elevated surface productivity at ~635 Ma. Subsequent migration of Laurentia to occupy mid‐high latitude in the southern hemisphere is represented in Figure 8b, however Li et al. (2013) caution that this is the reconstruction with least reliability. Occasional, short‐lived oxia recorded within the overlying upper Sheepbed, June Beds and Blueflower formations may be a product of deposition above the oxycline and/or an effect of lateral transport of subjacent oxygenated bottom water currents, similar to those which are inferred to have dominated the depositional environment of the distal Isaac Formation.

Both Avalonia and the EEP, which shared a similar latitudinal position to Laurentia at ~580 Ma, show contrasting evolution from ferruginous anoxia towards dominantly persistent oxygenation of the deep slope environment as early as ~579–575 Ma (Canfield et al., 2007; Johnston et al., 2012; Pu et al., 2016). The migration of the EEP to occupy a mid‐high latitude position close to western Avalonia during the final ~40 my of the Ediacaran Period alongside their corresponding shift towards more stable oxygenation, may reasonably lead to the tentative suggestion of a similar mechanism. The stable oxygenated conditions accompanying deep marine deposition of the Conception and St John Groups and neighbouring Vychegda and Redkino Formations require a well‐established hydrographic mechanism. Cooling of surface water in this region may have stimulated density‐induced deep‐water development in the aftermath of the regional Gaskiers deglaciation (Laflamme et al., 2013; Li et al., 2013). Under these conditions, oxygen‐rich water from the well‐mixed surface ocean would be drawn to depth in a similar manner to present‐day North Atlantic deep‐water formation (Broecker, 1997). Importantly, deep‐water formation in the modern ocean is seen to be location specific and dependent on factors including local bathymetry, freshwater input and evaporation (Broecker, 1997; Bruce, 1983). Whilst the key conditions conducive to deep‐water formation continued to characterise the vicinity of Avalonia and the EEP, cratonic positioning of the Laurentian passive margin likely favoured a regime of continued upwelling.

The Nama basin occupied a mid‐high latitude position freely connected to the narrowing Brazilides Ocean in the southern hemisphere in the final 10–12 million years of the Ediacaran. Mixed carbonate‐siliciclastic deposition in a ramp environment, occupying shallower levels of the water column than sections of Avalonia and the EEP, alongside surrounding assembly of Gondwanaland may have restricted development of effective deep‐water formation and maintained continued stratification with ferruginous anoxic depths (Figure 7d). A transition from dominantly anoxic and ferruginous water column conditions during deposition of the Kuibis Subgroup to long‐lasting oxia of the Urusis Formation, particularly of the Witputs sub‐basin, may also suggest a progressive deepening of the oxycline towards the Ediacaran‐Cambrian boundary. Meanwhile, contemporaneous closure of the intracratonic seaway, encroachment of the Arachania arc and differential sediment flux affecting the two sub‐basins may have led to the preservation of distinct geochemical signatures within a predominantly oxic shallow marine environment (Figure 7e). The stratified redox model proposed for the Arroyo del Soldado Group (Aubet et al., 2012; Pecoits, 2010) is consistent with that described from the Nama Group. However, with poor temporal constraint on deposition of the Yerbal and Polanco Formations, difficulty remains in determining stratigraphic equivalence between fossil‐bearing units of these two Groups.

In summary, whilst a gradual increase in global ocean oxygenation may have occurred during the Ediacaran Period (e.g., Chen, Ling, et al., 2015; Kendall et al., 2015), cratonic positioning likely influenced mechanisms for local oxygenation resulting in regions characterised by continued dominance of anoxia.

### 5.2 Controls on biotic distribution

#### 5.2.1 The distribution of biomineralising biota

Benthic *Sinotubulites* and *Cloudina* recognised across the Yangtze Block dominantly outcrop in shallow marine carbonate facies of the Gaojiashan and Beiwan members (and equivalents) of the middle and upper Dengying Formation (Figure 7c). However, specimens of *Sinotubulites* are also noted from deeper ramp limestones deposited above storm wave base of the Gaojiashan‐equivalent Shibantan Member (Cai et al., 2014). During deposition of the Dengying Formation, proxy evidence supports a temporarily oxic shallow shelf (Duda et al., 2014; Ling et al., 2013) which perched above a dominantly anoxic deeper water environment within which periods of euxinia may have been detrimental to calcification. Dominant anoxia and encroachment of the euxinic wedge into shallow inner platform environments typified the water column during deposition of the underlying Doushantuo member IV, which may have restricted colonisation of calcifying communities along the shelf and within the anoxic intrashelf basins until intermittent ventilation during deposition of the middle Dengying Formation, potentially as a consequence of occasional deeper water mixing by mass‐flow events, permitted transient habitation by mat‐sticking *Cloudina* (Cai et al., 2014). Geochemical and palaeontological investigations of Dengying Formation shallow platform carbonates at Gaojiashan (Shaanxi Province) suggest that the observed transition from soft‐bodied to calcareous biomineralisation represented by successive appearance of *Cloudina* after *Conotubus* may have been related to an increase in continental weathering‐derived sulphate and alkalinity (Cui, Kaufman, et al., 2016). In this scenario, initially more oxidising conditions supported soft‐bodied and bioturbating communities, whilst subsequently enhanced continental weathering associated with elevated water column alkalinity and carbonate saturation not only led to reinforced water column redox stratification but may, alongside the advent of predation, have promoted the necessity for biocalcifying communities inhabiting shallow oxic waters through instigating the requirement for a mode of Ca removal from newly developed circulatory systems (Cui, Kaufman, et al., 2016).

Shallow, inner‐ramp carbonate and siliciclastic sediments of the Omkyk and lower Hoogland members of the Nama Group host *Cloudina* and *Namacalathus* within transient oxic/manganous/ferruginous conditions attributed to the short‐lived incursion of anoxic deeper water (Figure 7d) (Wood et al., 2015; Tostevin, Wood, et al., 2016). However, water column anoxia induced via primary productivity and organic matter oxidation, as may have occurred in shallowest settings influenced by riverine nutrient influx, results in the by‐product of substantial dissolved CO_2_ and lowered *p*H conducive to enhanced CaCO_3_ dissolution (Sperling, Knoll, et al., 2015). Therefore, under lowered *p*O_2_ of the Ediacaran Period, long‐lived productivity‐induced anoxia as seen in modern OMZs is unlikely to have supported immobile, benthic, strongly‐calcifying organisms such as *Namapoikia*. Intervals associated with protracted oxia both throughout, above and below biomineralising fossil horizons, in shallow to mid‐ramp settings are accompanied by thicker walled and larger individuals reflective of the ease of metabolically demanding calcification within these stable oxic, oligotrophic, carbonate‐saturated waters (Wood et al., 2015).

Partial redox reconstruction of the terminal Ediacaran Arroyo del Soldado Group, Brazil (Figure 6c) lends further support to this proposed biotic response, with *C. riemkeae* recorded from the upper Yerbal Formation bracketed by oxic siliciclastic deposits (Frei et al., 2013; Gaucher & Poiré, 2009; Gaucher & Sprechmann, 1999). However, further high‐resolution geochemical sampling within the Arroyo del Soldado Group is required within a palaeontological and palaeo‐ecological remit to support protracted oxygen stability as a prerequisite for extensive calcification. Future integrated studies incorporating sections of the Upper Miette Group (Rocky Mountains), Wood Canyon (California), Dengying Formation (Yangtze Block), Itapucumi Group (Paraguay), Yudoma Group (Siberian Platform), lower Ara Group (Oman), Puerto Blanco Formation (Mexico), Bambuí and Corumbá Groups (Brazil), and Ibor and nivel de Fuentes Groups (Spain) may help establish this as a globally identifiable condition (Corsetti & Hagadorn, 2000; Cortijo et al., 2010; Cui, Kaufman, et al., 2016; Hofmann & Mountjoy, 2001; Hua et al., 2003; Sour‐tovar et al., 2007; Warren et al., 2014; Zhuravlev et al., 2012).

##### Metazoan reefs

The only documented occurrence of active reef‐building by *C. hartmannae* and *C. riemkeae* alongside dyke‐dwelling *Namapoikia* is recorded within mid‐ramp positions typified by inferred persistent oxygenation of the Nama Group, Namibia (Penny et al., 2014; Wood et al., 2015; Tostevin, Wood, et al., 2016). Prerequisite conditions for effective, long‐lived reef building today include stable substrate, low sediment influx, readily available Ca^2+^ and ${\text{CO}}_{3}^{-}$ ions and relatively well oxygenated conditions above the contemporaneous carbonate compensation depth (James & Jones, 2015). Under these ideal conditions Ediacaran *Cloudina* reefs grew and there is some evidence for confinement of reef growth to exclusively oxic intervals of the mid‐ramp at Driedoornvlagte. Establishment of a pelagic‐benthic link was likely enabled in such ecosystems through proficient suspension feeding by *Cloudina* and *Namacalathus*. This may have resulted in rapid and effective redistribution of organic matter to depth thereby supporting a model of biological ventilation of shallow and mid‐depth environments via reduced oxygen consumption in surface waters towards the end of the Ediacaran Period (Butterfield, 2009; Lenton et al., 2014; Meyer et al., 2016).

#### 5.2.2 Soft‐bodied macrobiota

Whilst some regions indicate a distinct trend towards local oxygenation accompanied by first fossil appearance of complex multicellular eukaryotes (e.g., Avalonia, the EEP and the Kalahari Craton) assemblages including the Avalon assemblage of the Laurentian passive margin, and Lantian and Miaohe biota of the Yangtze Block, are seen to have occupied dominantly anoxic ferruginous bottom water environments. Therefore, if dissolved oxygen above a threshold concentration was necessary to support such communities, benthic colonisation occurred during short‐lived oxic episodes indistinguishable by current proxy methods.

Potential benefits of inhabiting an environment prone to dissolved oxygen depletion may be associated with nutrient demand. For instance, upwelling or continental nutrient delivery and elevated primary production might conceivably have favoured organisms such as rangeomorphs which may have fed via osmotrophic absorption of labile DOC or active fluid endocytosis during periods of less active vertical mixing (Laflamme, Xiao, & Kowalewski, 2009).

With the exception of *Eoandromeda* which is recorded from ferruginous deposits of the Miaohe member, soft‐bodied fossils considered to represent probable Metazoa including *Thectardis* and *Kimberella* have so far been noted only from stratigraphic sections known to have been deposited beneath oxygenated bottom waters. This may be considered to support the inference of relatively high physiological oxygen demand suggested for motile *Kimberella* (Sperling, Knoll, et al., 2015).

#### 5.2.3 Benthic sulphide stress: Yangtze Block

Semi‐restricted intrashelf basins of the Yangtze Block were prone to protracted anoxic intervals typified by free water column hydrogen sulphide and quantitative trace metal drawdown (Li et al., 2010; Och et al., 2015; Sahoo et al., 2016). Whilst localised hypoxia in the modern ocean reduces macrofaunal biodiversity, the additional deleterious effects of elevated H_2_S_aq_ on aerobic benthic communities is also well documented, with accelerated mortality during anoxic periods in the presence of H_2_S_aq_ due to the enzymatic disruption of oxygen carrying cytochrome *c* oxidase at the terminus of the mitochondrial electron transport chain (Vaquer‐Sunyer & Duarte, 2010). The toxic effects of hydrogen sulphide are experienced by bivalve and annelid species in the modern ocean at μmol/L concentrations below those expected to result in quantitative scavenging of Mo such as that recorded from black shales of the Doushantuo Formation and overlying terminal Ediacaran and Cambrian formations of the Yangtze Block (Vaquer‐Sunyer & Duarte, 2010). As such, under conditions of elevated aqueous H_2_S, body plans which exhibit higher surface area to volume ratios are expected to suffer greater losses (Sperling, Knoll, et al., 2015).

Extensive benthic sulphide stress of Yangtze Block environments following transgression and flooding of intrashelf basins during deposition of Doushantuo Member IV is likely to have been severe, with long‐lived “patchy” water column euxinia persisting into the early Cambrian when sessile benthic communities were likely restricted to inner shelf platform settings (Och et al., 2015). Despite the extreme environmental conditions, a diverse macrofaunal assemblage developed within basins of the Yangtze Block. Repetitive flooding by reducing waters may have been accompanied by repeated local community die‐off, this may also have irrigated shallower depths through nutrient recycling from the deeper water environment in preparation for recolonisation during subsequent ventilation. It may be reasonable to assume that, although we know little about the physiological requirements of the Miaohe biota, fossil representatives of probable animals such as *Eoandromeda* may have suffered considerably under anoxic conditions with elevated H_2_S. Interestingly, iron speciation data of lower fossiliferous units of the Doushantuo member IV at the type locality of Miaohe (Hubei) show a dominance of ferruginous anoxia, with elevated proportions of pyrite indicative of euxinia restricted to overlying shales devoid of fossils (Li, Planavsky, et al., 2015). Similar high‐resolution geochemical sampling may benefit physiological discussions of the fossiliferous Lantian Formation. Whilst the early Cambrian saw continued euxinia in environments of the Yangtze Block, this may have gradually given way to a less stressed inner shelf environment with elevated water column H_2_S largely restricted to the lower slope and basin (Feng et al., 2014; Och et al., 2015; Wang et al., 2012; Yuan et al., 2014).

## 6 Conclusions

Through development and application of varied geochemical proxies, the past decade has witnessed a revolution in our understanding of global and local redox heterogeneity which accompanied the evolution of ecosystems containing potential candidates for the earliest animals. Although earlier studies suggested that oxygen stabilisation may have characterised the global ocean as early as the late Ediacaran (Canfield et al., 2007; Johnston et al., 2012), continued local redox heterogeneity is evident from multiple shallow to deep marine environments well into the Cambrian. Whilst increase beyond a low *p*O_2_ concentration threshold within the shallow marine environment may have enabled a step‐change in complexity of middle Ediacaran ecosystems, in some areas this facultative threshold may have been surpassed only transiently. Currently available bulk rock redox proxy methods are unable to resolve short‐term oxygenation which may have permitted opportunistic colonisation of the substrate by organisms with higher oxygen demand. However, high‐resolution geochemical sampling conducted in a comprehensive palaeontological and palaeoecological framework, despite the inherent complications associated with taphonomic and facies biases, enables appreciation of changing provincial ecosystem structure together with the extent of accompanying local water column oxygenation through the Ediacaran (Liu et al., 2015; Sperling, Carbone, et al., 2015). The importance of placing such studies in relative sea‐level and palaeogeographic framework will enable appreciation of the nuances of marine redox heterogeneity which characterised environments on the kilometre scale, similar to those that exist in modern shelf‐to‐basin environments.

Whilst a decrease in the volumetric proportion of euxinic mid‐depths during the Cryogenian is suggested to have removed a toxic barrier to evolutionary diversification of aerobic eukaryotes (Guilbaud et al., 2015), the oxygen concentration of shallow waters was likely sufficient to support evolution of the earliest Metazoa represented by crown group demosponges in the Cryogenian (Sperling, Halverson, et al., 2013). Subsequent environments of the Ediacaran witnessed the proliferation of probable animals which appear to have opportunistically colonised habitable substrate. This may, in part, have been defined by the availability of dissolved oxygen under oxic/dysoxic conditions, with the greatest diversity permitted in environments subject to effective oxygenation. Once developmental barriers to biomineralisation were surpassed, local ecosystem feedback associated with biological ventilation of the water column may have initiated towards the end of the Ediacaran and incorporated efficient filtration of rapidly sinking, large organic particles via suspension feeding within high surface area to volume ratio, multitiered reefal ecosystems, alongside substrate bioturbation and efficient phosphorus retention (Lenton et al., 2014; Penny et al., 2014; Wood & Curtis, 2015).

The Ediacaran Earth saw extensive cratonic migration during the formation of Gondwana and associated alterations in available niche space. The geographic positioning of palaeoenvironments within the global ocean, alongside their relative palaeodepth, likely influenced mechanisms for changing redox in open shelf environments. When viewed together, Ediacaran geochemical, palaeogeographic and palaeoenvironmental data suggest local ecosystem dynamics constrained by parameters including dissolved oxygen availability, nutrient provision, stable substrate for colonisation and the evolution of predation.

Continued environmental proxy development and utilisation in high‐resolution biostratigraphic and palaeoecological studies across shelf‐to‐basin transects may aid in clarification of compelling issues associated with Ediacaran ecosystem development. Particularly, the development of novel geochemical proxies which record instantaneous transitions sensitive to minor changes in dissolved oxygen concentration may shed light on the necessary oxygen requirements for shallow marine colonisation at ecologically meaningful timescales in the earliest calcifying invertebrate communities.
